# Social judgments at the intersection of class and gender across cultures

**DOI:** 10.1371/journal.pone.0338029

**Published:** 2026-02-18

**Authors:** Marie Isabelle Weißflog, Lusine Grigoryan, Wilhelm Hofmann

**Affiliations:** 1 Department of Social and Environmental Psychology, Ruhr-Universität Bochum, Bochum, Germany; 2 Department of Psychology, University of York, York, United Kingdom; 3 Partner site Bochum-Marburg, German Center for Mental Health (DZPG), Bochum, Germany; Pacific Lutheran University, UNITED STATES OF AMERICA

## Abstract

To address social injustice, it is crucial to understand the intersecting social dimensions that contribute to it, such as gender, race, and class. While intersections of race and gender are well-studied, class remains underexplored in social psychology. This research investigates how class (measured by education, income, and occupational status) and gender influence interpersonal attitudes regarding likability, respect, and social distance across different cultures. We present results from factorial survey experiments in eight countries (Armenia, Australia, Brazil, Germany, India, Russia, UK, US) with different gender norms and inequality levels. High education and income influenced attitudes towards women (vs. men) more positively, and low income and occupational status influenced attitudes towards men (vs. women) more negatively. In countries with more conservative gender norms, these differences were stronger. General inequality also impacted status- and gender-based attitudes. Our findings demonstrate that gender and class interact differently across cultures, contributing to discourses on intersectionality and informing social equality and policy interventions.

## Introduction

Social inequality and injustice are based on group memberships, like gender, race/ethnicity, and class. For example, women and gender minorities experience workplace discrimination [1,2], and restrictive gender roles affect people of all genders in intimate relationships [3]. Similarly, racism harms people’s health and healthcare access [4–6], and racial/ethnic minorities experience workplace discrimination [7]. People from lower socioeconomic backgrounds face exclusion in settings including housing, health care, and education [8–10]. These issues are typically examined along individual dimensions of gender, race/ethnicity, or class [11]. This monolithic approach, however, risks overlooking how these dimensions interact in shaping social realities [12,13]. For instance, social psychologists have begun to consider intersections between race/ethnicity and gender, for example gender-race-stereotypes [14–16], to better understand complex discrimination patterns, like backlash against female leaders of different races [17]. Despite this progress, the intersection of social class and gender has received comparatively less attention. The present article contributes to filling this gap by examining the interplay of social class and gender, two key dimensions of social inequality. To develop our hypotheses, we first review existing research on social class and then turn to intersections of class and social status with gender.

### Social class and (socioeconomic) status

In recent years, researchers have advanced conceptualizations [18–21] and empirical studies of social class and socioeconomic status [22–25]. Both class and social or socioeconomic status (SES) describe how individuals and groups are positioned in a social context and, as a result of their position, have different degrees of access to economic, social, and cultural resources [26]. Definitions of class and status vary; class has traditionally been defined based on labor relations in which ownership and control of productive assets enables some groups and individuals to exert control over others and exploit their surplus labor [27–29]. However, class has also been analyzed as a form of culture [19–21], with individuals learning distinct norms and values through socialization in their class environments [30]. Social status, on the other hand, has been used to describe an individual’s or group’s relative social position more broadly as the value or importance assigned to them based on stratifying group memberships and characteristics, such as race/ethnicity, gender, or skills [31], including characteristics that are also associated with class position, such as occupation. Some theories posit status as purely symbolic [32], others define it as a symbolic yet inextricable aspect of class [33]. Socioeconomic status represents social and economic circumstances of an individual or group and their relative position, drawing on a variety of indicators commonly including occupation, income, and education [34]. We acknowledge that definitions of social class and (socioeconomic) status are contested and vary across disciplines and theoretical frameworks. Depending on the specific definition, our variables of interest – occupation, income, and education -, could to varying degrees serve as indicators of both status and class. We therefore use the terms “status”, “SES”, and “class” interchangeably in this manuscript, rather than prescribing a specific mapping of occupation, income, and education to class, SES, and/or status according to one specific definition of these constructs. However, as outlined above, these concepts are not necessarily equivalent and there can be valid reasons for further differentiation depending on theoretical framework and research questions.

Across different conceptualizations of social inequality, there is ample evidence of its manifestation in and impact on individuals, groups, and societies. Poor people are disadvantaged in multiple domains like housing, health care, and education [8–10,35]. Class influences health [36–38] and well-being [39–41]. It shapes social perception [22,23,25,42–44] and behavior [24,45]. Importantly, high class is not simply the absence of low-class disadvantages, but distinctively shapes cognition [42,46], behavior [24,46], and perception by others [47,48]. Many people across cultures hold ambivalent warmth and competence stereotypes about rich and poor individuals [48–50]. Access to resources associated with class is often operationalized through socioeconomic markers like education, income, and occupation. However, these markers correlate only moderately [51], and predict different outcomes [52]. For instance, income is associated with current access to economic resources, preferences for self-sufficiency [53], and political attitudes opposing economic redistribution [54]. Education, on the other hand, is linked to cultural capital, prestige [55], trust and political interest [56], and liberal political attitudes [54]. Occupations differ in perceived social status, and people commonly build social connections and friendships with others who work in a similar sector and/or occupation with similar status [28,32]. Similarly, a person’s income, education, and occupation may each uniquely influence how others perceive them. For example, someone’s occupation impacts how others perceive them, depending on general stereotypes about the occupational group – people tend to assume that someone working in a prestigious occupation would be assertive and competent [57]. Capturing the nuances of each SES marker independently enables a deeper understanding of the relationships between SES and various outcomes.

### Intersection of gender and class

Whereas there is an extensive literature on gender and social class, respectively, the *intersection* of gender and class remains underexplored. Investigating the intersection of social class and gender is crucial both regarding how perceivers judge targets with various combinations of gender and class, which may result in stereotyping and discrimination, as well as how targets are treated, i.e., the resulting positive or negative psychological experiences from social interactions. Whereas the primary focus of this research is on perceiver effects (i.e., impression formation), these two aspects are connected, as social judgments may give rise to experiences of discrimination [8,58]. Gender is linked to status beliefs – with men generally expected to have higher status than women [59]. Status is central to agentic masculine stereotypes and role expectations, as opposed to communal feminine stereotypes and roles [60–62]. Men are expected to embody traits that are associated with high status, like agency and independence, and avoid displaying traits associated with low status, like weakness and shyness [61]. Women are not necessarily expected to embody traits signaling low status, but to avoid displaying traits associated with high status.

Existing research on intersections of gender and status is predominantly based in organizational contexts, where people often perceive women as unfit for leadership positions associated with traditionally masculine traits [63,64]. Perceivers respond negatively to women who violate role expectations by occupying or striving for high-status positions, especially when this includes exhibiting dominant behaviors [59,65–73]. Similarly, people judge men more negatively who violate role expectations by behaving modestly [73] or being stay-at-home fathers, while working mothers are likewise judged more negatively than stay-at-home mothers [74].

**Fig 1:**
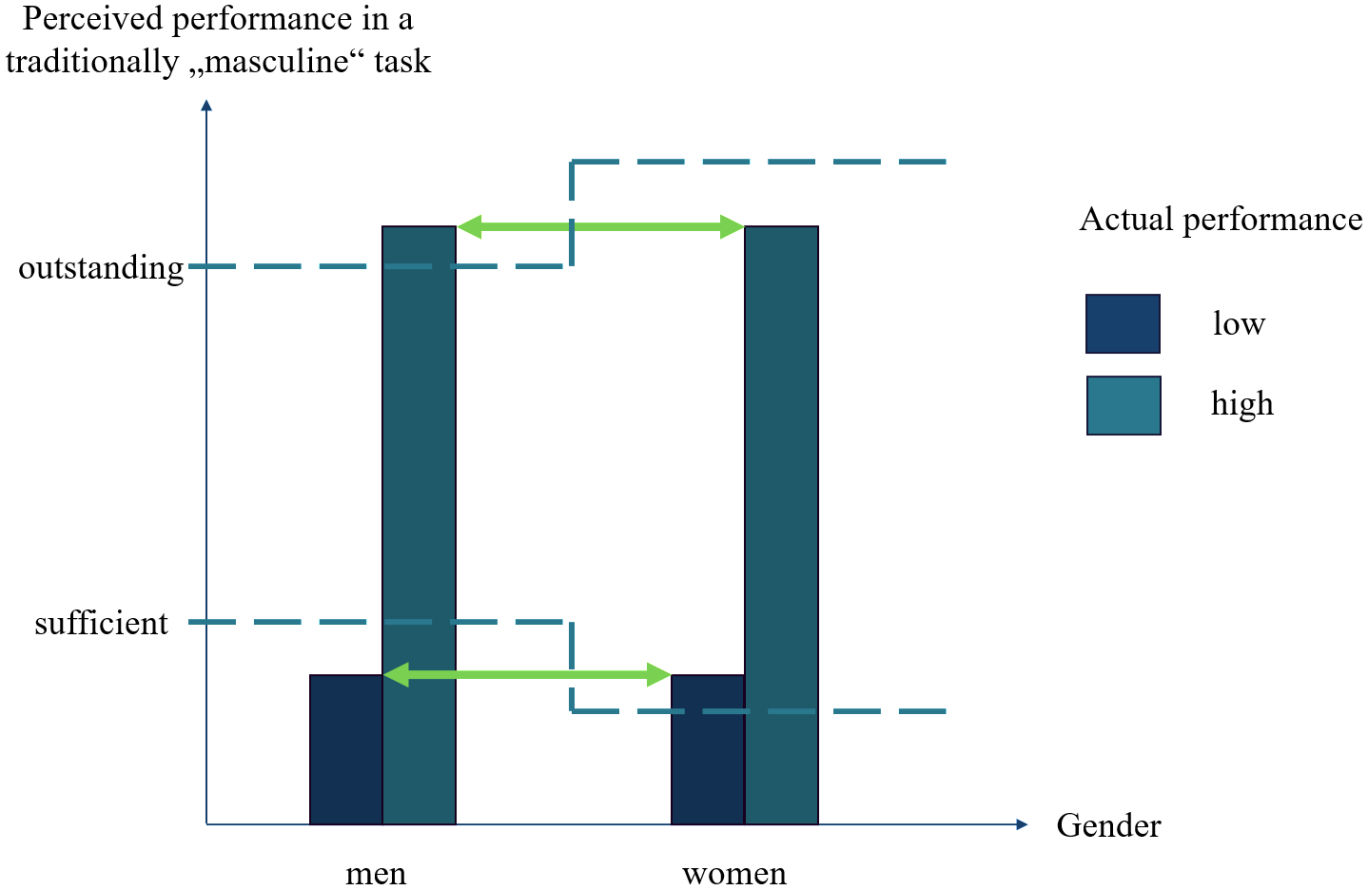
The shifting standards model. The figure visualizes the shifting standards model. The x-axis shows gender represented by the two groups of women and men. The y-axis shows perceived performance in a traditionally “masculine” task. The colored bars show actual performance, with darker bars indicating lower performance. The dotted lines show thresholds for perceived sufficient and outstanding performance, respectively. Note that these thresholds differ for women vs. men, with a lower threshold for women’s performance to be perceived as sufficient, but a higher threshold for their performance to be perceived as outstanding. This can lead to different judgments of the same performance level in women vs. men, in that low performance or status is judged more acceptable for women, while high performance or status is judged more positively for men.

Performance evaluations often apply shifting standards based on group memberships and associated stereotype-based expectations [75]. Women, stereotyped as less competent than men, have lower thresholds to meet minimum competence standards (i.e., the minimum ability needed to perform a skill) in stereotypically masculine skills [76–78], but higher thresholds to be considered excellent [79]. When in a leadership position, women are perceived as less legitimate than men and are subject to double standards that penalize them for showing dominant emotions like anger or pride, which are deemed acceptable in men [80–82]. This may also apply to class and status standards, with low-income women potentially evaluated more positively than low-income men, and high-income men more positively than high-income women. Fig 1 visualizes the shifting standards model.

The precarious manhood hypothesis highlights the fragile nature of masculinity, where a man’s status as “manly” is not a given but something that must be continually earned and is easily lost or taken away in the eyes of others [83]. This concept intersects with social class, illustrating how the pressure to uphold masculine ideals is particularly intense among men facing status threats. The link between masculinity and social power means that when a man loses economic or occupational status, it can also be perceived as a loss of masculinity [83]. Therefore, men might be more affected by status threats compared to women, fearing and facing social judgment for failing to conform to masculine role expectations [84]. This intersection underscores the complexity of how gender and class dynamics shape individual experiences and social attitudes, as in men who are judged harshly when they fail to conform to traditional masculine roles, such as being the primary breadwinner.

The subordinate male target hypothesis (SMTH) suggests that direct intergroup hostility is mainly directed towards men of subordinate groups [85,86]. Therefore, lower-class men might face more negative judgment than women. Intersectional invisibility theory posits that individuals with multiple subordinate identities, such as working-class women, are non-prototypical for each of their overlapping groups and may experience distinct disadvantages [87]. This invisibility can shield them from direct discrimination but also results in a lack of representation and influence. In line with both SMTH and intersectional invisibility, lower-class men report more direct discrimination experiences than women [88]. Poor men are also stereotyped more negatively and blamed more for their poverty than women [89]. However, multiply marginalized women also face distinct disadvantages – for example, working-class women face more victim-blaming after gender-based mistreatment than middle-class women [90,91].

In summary, compared to research on social status and gender in organizations, e.g., regarding leadership or performance, social judgments of women and men of different class backgrounds remain understudied. The aforementioned frameworks from the organizational and intergroup literature led us to suggest, however, that social class and gender may intersect much more dynamically than typically assumed. These intersectional dynamics are essential to a more comprehensive understanding of social stratification and its effects on individuals’ lives [92]. **Table 1** summarizes the theoretical frameworks discussed so far and expected outcomes for women and men at different class positions based on each framework.

**Table 1:** Summary of theoretical frameworks and expected outcomes at intersections of gender and class.

Framework	Key ideas	Implications for gender-class judgments
**Status beliefs & gender role expectations**	According to masculine gender roles, men are expected to be agentic and have high status According to feminine gender roles, women are expected to be communal Women in high-status roles and men in low-status roles violate role expectations and can face backlash and negative judgment	Status impacts attitudes towards men via low-status proscriptions and high-status prescriptions, and for women via high-status proscriptions High SES valued more in men and may even be perceived negatively in women Low status judged more harshly in men
**Shifting standards theory**	Performance is evaluated relative to group performance stereotypes For groups stereotyped to perform well on a task or criterion, the threshold to be considered sufficient is higher, but the threshold for being considered outstanding is lower For groups stereotyped to perform less well on a task, the threshold to be sufficient is lower, but the threshold for being outstanding is higher Women often stereotyped as less competent and powerful than men, so they may have lower minimum thresholds to be considered sufficient in status, but higher thresholds to be considered exceptional in status	Low-status women judged more positively than men (lower threshold for “sufficient”) High-status men judged more positively than women (higher threshold for women to be “outstanding”)
**Precarious manhood hypothesis**	Masculinity is tied to social power and economic/ occupational status, which is “hard to earn, easy to lose” Status loss thus also threatens men’s masculinity	Low-status men face amplified negative judgments Status especially important for attitudes towards men
**Subordinate male target hypothesis (SMTH)**	Direct intergroup hostility mostly directed from men from superordinate groups at men from subordinate groups.	Lower-status men more likely targets of negative attitudes and direct discrimination than women.
**Intersectional invisibility**	People with multiple subordinate identities (e.g., working-class women) are non-prototypical for each group Overlooked as members of either group and/or stereotyped differently Invisibility may shield from some direct hostility but limits influence and representation.	Low-income women experience less overt negative judgment than men, but potentially other unique disadvantages

### Gender and class in cultural context

The intersection of gender and class also needs to be viewed in a cultural context: Power structures around class and gender and associated social norms are embedded in the respective culture, shaping stereotypes and attitudes. For example, gender stereotype content varies depending on cultural values, with men being stereotyped closer to the respective cultural ideal [93]. Stereotypes can also change over time in the same cultural context, reflecting shifts in social roles [94], which interact with cultural beliefs about gender [95]. While there is less cross-cultural research on perceptions of class, experimentally manipulated context inequality has been shown to heighten class stereotypes [47,96]. The way education and income influence one’s social status and personal identity can also differ based on cultural norms and values [97] suggesting that perceptions of both gender and class vary by context. Cultural narratives around social mobility and status can influence how individuals from different classes are perceived [50,98], which may also interact with their gender. Additionally, research suggests that precarious manhood beliefs are uniquely associated with patriarchal social structures at the country level. In countries where men have significantly more political power, control over resources, and better health outcomes than women, the perception that manhood must be earned and is easily lost is more prevalent [99]. In conclusion, there is considerable evidence to suggest that the intersectional influence of gender and social class on judgments of others can be influenced by the cultural context in which these judgments are made.

### The present research

This paper investigates how people evaluate others based on gender and class intersections, exploring also cultural (i.e., country-level) differences and potential explanatory variables. In a factorial survey experiment, we manipulated social dimensions, including class markers (education, income, occupation) and gender (women, men). This approach allows us to examine class markers separately, since they do not correlate perfectly and predict different outcomes [51,52]. We examined participants’ attitudes towards vignette targets depending on gender, education, job, and income across eight countries to improve generalizability and explore the role of context.

We hypothesized that higher status would predict more positive attitudes for men more strongly than for women, since status-associated agentic qualities are more important for men’s role expectations. In line with intersectional invisibility and SMTH, we expected low-status men to face more negative attitudes compared to low-status women. According to shifting standards theory, we also expected low-status women to be more positively evaluated than low-status men, and high-status men to be more positively evaluated than high-status women. The main analysis was preregistered on https://aspredicted.org/F61_RWJ. Originally, we planned to examine both overall attitude score and the dimensions of respect and liking separately. We only focus on the overall attitude as an outcome in this paper, but report the results for the two dimensions separately in supplementary materials available on OSF. This approach was supported by high correlations between attitude items (.69 −.85), implying that a combined analysis would still adequately reflect participants’ attitudes. See supplementary material for separate analyses for liking and respect. Results were mostly the same regarding our hypotheses. Where there were differences between liking and respect, this will be noted in the results.

Additionally, we explored if country context moderates these relationships, focusing on country-level gender norms, gender inequality, and general inequality. This comprehensive approach aims to fill the gap in understanding how gender and class intersections influence interpersonal attitudes across different cultural contexts.

## Methods

All materials, including supplementary materials, data, and code are available on OSF: https://osf.io/6wjxd/?view_only=b9d29c36d3d14c91abc24ab016e79630

### Participants

We included 2,714 participants (N_obs_ = 26,887) from eight studies in eight countries: Germany (N = 301, N_obs_ = 3,003), UK (N = 301, N_obs_ = 2,998), US (N = 307, N_obs_ = 3,040), Russia (N = 524, N_obs_ = 5,036), Australia (N = 359, N_obs_ = 3,590), Armenia (N = 311, N_obs_ = 3110), Brazil (N = 282, N_obs_ = 2,820), and India (N = 329, N_obs_ = 3,290). Sample sizes were based on recommendations for multilevel models [100,101]. The data from Russia were originally collected by one of the authors in 2015 and the data from Australia, Armenia, Brazil, and India by the same author in 2017 to test different hypotheses [102–105]. The anonymized datasets are available on OSF and were accessed on November 29 (Russian sample) and December 5 (remaining samples), 2022.

These four countries were originally selected to represent different configurations of extreme scores on social inequality and acceptance of cultural diversity (ACD) based on data from the World Values Survey, wave 6 [106] and the Human Development Report 2015 [107], with Australia representing relatively low inequality and high ACD, Armenia representing low inequality and low ACD, Brazil representing high inequality and high ACD, and India representing high inequality and low ACD. For more details on country selection, see [105]. We collected the data from Germany, UK, and US between March 31 and September 12, 2022 for a new project which the current paper is a part of. We ensured each sample included members of all social groups represented in the vignettes. S1 Table shows sample demographics.

Fifteen participants under 18 years old were excluded from the Russian sample, and two participants due to technical errors. We excluded no other participants.

### Design and procedure

All studies employ factorial survey designs, which combine experimental flexibility, high external validity, and low social desirability effects [101]. Factorial surveys have been used to examine interpersonal attitudes [108–110] and prejudice [111–113].

Participants were randomly assigned to one of several blocks with 10 vignettes each. The total number of blocks in the US, UK, and Germany was six, in Armenia, Australia, Brazil, and India three, and in Russia, ten. Vignettes described people whose characteristics systematically varied on eight to nine social dimensions, depending on country, across two to six levels, depending on the dimension. In the two original studies, the authors selected dimension levels considering functional equivalence across countries, supported by interviews with local experts on intergroup relations. 8–11 social scientists with regional intergroup expertise from each country participated in semi-structured online questionnaires to determine which dimensions of social categorization were most salient in their respective countries. They were asked about the perceived importance of several preselected dimensions, and any important dimensions the researchers had not mentioned. In the US, UK, and Germany, we used the same levels across countries for income and job due to cultural similarity. In the previous cross-cultural study (Armenia, Australia, Brazil, India), authors chose categories that, based on expert interviews, were functionally equivalent, but varied in wording (e.g., low-skilled worker in Brazil vs. tradesperson in Australia). The Russian study used levels capturing salient social categories in Russia. For example, since most Russians have tertiary degrees, they are a weaker status marker than in countries where tertiary education is less common. Therefore, the authors used PhD as highest education level in Russia. Job levels were low-skilled worker, skilled professional, and highly skilled specialist. We combined the latter two into a “professional” level. Since unemployment was not included in Russia, there were only two job levels. Despite identifying functionally equivalent levels across countries, potential differences in perception of levels should be kept in mind when interpreting the results. Table 2 shows specific dimensions per country and their levels.

**Table 2:** Factor levels by dimension and country.

Variable	Levels							
	***Germany***	***UK***	***US***	***Australia***	***Armenia***	***Brazil***	***India***	***Russia***
**Gender**	------------------------------------------------------------------------------------- Woman ------------------------------------------------------------------------------------							
	--------------------------------------------------------------------------------------- Man --------------------------------------------------------------------------------------							
**Income**	Above average			Better off than the average Australian	----------------------------- Rich ---------------------------			
	Average			On a par with the average Australian	---------------- Has an average income ---------------			
	Below average			Worse off than the average Australian	---------------------------- Poor ----------------------------			
**Job**	Professional			Professional	Skilled professional		Professional	Highly skilled specialist/skilled professional
	Manual worker			Tradesperson	Low-skilled worker		Laborer	Low-skilled worker
	----------------------------------------------------- Unemployed ------------------------------------------------------							
**Education**				Completed high school to year 10	Completed high school	Completed primary school	Studied up to primary school	No higher education
				Completed vocational training	Attended college	Completed high school	Studied up to high school	Has higher education
				----------------------------------- Has a university degree ----------------------------------				Has a Ph.D.

Dark grey fields indicate dimension or level was not included in this sample.

**Fig 2:**
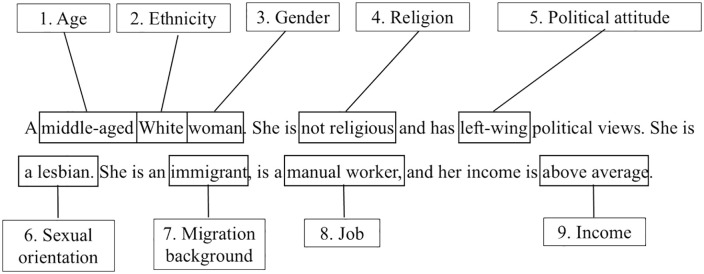
Example stimulus used in the UK/US, with manipulated dimensions highlighted.

In the US/UK/Germany, we manipulated nine dimensions: gender, migration background, political attitude, sexual orientation (2 levels each), income, job, religion, ethnicity, and age (3 levels each). The factorial design was created using SAS [114]. From the vignette universe (all possible combinations of variable levels, n = 3,888), we sampled n = 60 vignettes for a D-efficient fractionalized design. The D-efficiency coefficient, which indicates design strength (balance and dimension orthogonality), was 92.5 (standard prediction error 0.89) – sufficient power to detect effects of dimensions on attitude [101]. The vignette sample was split into 6 blocks of 10 to prevent participant fatigue. Fig 2 shows an example stimulus from the UK/US, highlighting the nine manipulated dimensions.

In the US, UK, and Germany, we did not exclude any vignettes that participants might perceive as implausible. However, in the remaining countries, some implausible combinations (e.g., being a professional and poor in Brazil) were excluded before creating a design with the remaining vignettes. Excluding implausible combinations from a factorial design necessarily reduces the design efficiency, and one of the strengths of these designs which is the examination of all potential combinations of dimensions to better understand each dimension’s specific impact [101]. On the other hand, implausible vignettes may influence participants’ answering behavior in unwanted ways [101,115]. For example, participants may either stop paying attention to the dimensions causing the implausibility – when confronted with an unemployed person with high income, they may base their reaction solely on the other factors than income and occupation that are not as conflicting. Or instead, they may overly focus on the conflicting dimensions of income and occupation in an effort to make sense of them. However, when comparing the impact of each dimension on attitudes between the waves of data collection where implausible combinations were or were not excluded, implausible vignettes did not eliminate or drastically increase the effects of the dimensions involved. This suggests that implausible combinations were not frequent or extreme enough to invalidate participants’ responses.

This paper focuses on gender, job, income (manipulated in 7 out of 8 countries), and education (in 5 out of 8 countries). Job, income, and education are related, but not perfectly correlated, and predict different outcomes [51,52]. Including all three allows to differentiate dimension-specific effects. Education and job levels represent specific demographic categories, e.g., college education, while income represents relative status. Thus, our design covers both aspects of class [116–118]. Each level of each dimension occurred balanced across other levels and dimensions. This orthogonality allows to only analyze a subset of dimensions without bias when excluding others. It also allows to disentangle effects of dimensions that are often confounded in real-world contexts, as is the case with status markers like education and occupational status, as well as other characteristics linked to socioeconomic stratification, such as gender and race/ethnicity. By explicitly providing information about important social dimensions, and manipulating them independently from one another, we can greatly reduce the confounding effects that usually exist between these group memberships due to their factual and assumed correlations. For example, the explicit information about ethnicity in the vignettes ensured that participants would not imagine the person in the vignette as belonging to an ethnic group that they implicitly associated with a given socioeconomic status [119].

The US, UK, and Germany questionnaires also included measures of social group perception, identification, perceived discrimination, and mental health (see OSF for details). Participants evaluated the vignettes first, so vignette ratings were unaffected by other measures.

Design details and additional measures included in the earlier studies are presented in the respective publications [102–105]. Participants gave written informed consent before participating in the study. Each study complied with the respective university’s ethical guidelines, which waived ethics review for the studies conducted in Russia (Higher School of Economics, Moscow, Russia), and Armenia, Australia, Brazil, and India (Jacobs University Bremen, Germany). Data collection in the UK, US, and Germany was approved by the ethics committee of the faculty of psychology at Ruhr University Bochum, Germany.

Data were collected via the survey platform Qualtrics [120] in Russia, Germany, UK, and US, and via Unipark in Australia, Armenia, Brazil, and India. Participants were recruited online via recruitment platform Prolific [121], survey companies MSI (Germany) and Lightspeed (Australia, Brazil, India), the Turpanjian Center for Policy Analysis (Armenia), and social media and ethnic Diaspora online forums (Russia). We chose recruitment avenues based on availability in each country. In Germany, where it proved difficult to reach a balanced sample composition regarding our demographic criteria specified before via Prolific alone, we enlisted the survey company to specifically recruit participant groups underrepresented in the Prolific sample. For some hard-to-reach minority groups in Armenia and India, the survey was conducted as a computer-assisted interview instead. Participants in all countries except Russia and Armenia received monetary compensation via the respective panel providers.

### Measures

#### Attitude.

In Russia, attitude was measured with two items on a 10-point scale from 1 - “not at all” to 10 - “very much”: one social distance item “Would you like this person to be your neighbor?” and one likability item “Do you like this person?”. Spearman-Brown consistency was.92. In Australia, Armenia, Brazil, and India, attitude was measured with three items on a six-point scale from 1 – “not at all” to 7 – “very much”: “I like/respect/want to engage with this person.” (likability/respect/social distance, respectively). Cronbach’s α was.81−.92. In Germany, UK, and US, we used the same items, but a six-point scale. Cronbach’s α was.87−.92. Measures were z-standardized before combining them into a single measure (min: −2.29, max: 1.92).

#### Country-level variables.

All country-level data were drawn from the United Nations Development Programme’s (UNDP) Human Development Report 2021−22 [122]. All country-level variables were z-standardized for the analysis.

We used the Gender Social Norms Index (GSNI; [122] as an indicator of country-level gender norms. The GSNI consists of seven items from the World Values Survey [122,123], asking about gender bias on four dimensions: political (e.g., “Men make better political leaders than women do”), educational (e.g., “University is more important for a man than for a woman”), economic (“Men should have more right to a job than women”), and physical integrity (e.g., “Justifiable - For a man to beat his wife”). The GSNI is based on the latest available WVS data for each country as of the Human Development Report 2021–22, Data from India stemmed from wave 6 of the WVS (2010–2014), while data from the remaining countries stemmed from wave 7 (2017–2022). The GSNI indicates for each dimension which percentage of the population agrees with any of the biased statements. We calculated the mean across dimensions.

We used the Gender Inequality Index (GII; [122] to represent gender inequality. It includes inequality indicators on three dimensions: reproductive health (maternal mortality, adolescent births), labor market (women’s participation in paid labor), and empowerment (women’s parliamentary representation and secondary education). The GII is based on data up to 2021, including maternal mortality ratios in 2017, and adolescent birth rates, share of seats held by women in parliament, as well as differences in secondary education and labour force participation rates by gender, all in 2021.

For general social inequality, we used the Inequality in income and Inequality in education measures, based on the Atkinson inequality index [122]. The Atkinson inequality index ranges from 0 to 1 and is calculated as A = ε – g/μ, where ε is an inequality aversion parameter that determines the sensitivity to changes at the lower end of the distribution. The UNDP uses ε = 1. g is the geometric mean, μ the arithmetic mean of the distribution. Inequality of income is based on disposable household incomes, inequality of education on years of schooling, compiled and harmonized in international databases, such as the World Bank’s International Income Distribution Database [124]. A detailed description of the data sources is available in the UNDP’s technical notes on the Human Development Report 2021/22 [125]. We used the sum of income and education inequality scores as a general inequality score for each country. Higher values on the inequality indices reflect higher inequality.

### Analytical strategy

Analyses were conducted in R 4.1.2 [126] in the RStudio environment [127]. Packages used for data preparation were *haven* [128], *psych* [129], *readxl* [130], *tidyverse* [131]. For analysis, we also used *lmerTest* [132], *lme4* [133], and for visualization *patchwork* [134], *scales* [135] and *sjPlot* [136]. The independent variables are target’ gender, income, education, and job, and country-level gender norms, gender inequality, and social inequality. The dependent variable is attitude towards the target. Vignettes are nested within respondents, and respondents within countries, so we used multilevel regression. Individual-level variance of attitude (intraclass correlation, i.e., the ratio of the variance between participants to the total variance, or the correlation among vignette ratings of the same participant) ranged from.33 to.64, meaning that 33–64% of the variance could be attributed to differences in participants’ average attitudes.

We ran multilevel regressions for each dimension (education, job, income), using dummy coding with the middle level as reference to examine both effects of lower-than-average and higher-than-average status. Thus, we compared both low and high education levels to average education level, both unemployment and professional job to manual job, and both below-average and above-average to average income. Each model predicted attitude by the respective social class dimension and gender, and their interaction. Afterwards, we examined the effect of each country-level variable, respectively (GSNI, GII, inequality). We first added the respective country-level variable and two-level interactions with gender and the respective status dimension. Then, we added the three-level interaction between the respective dimension, gender, and the country-level variable.

We ran a number of robustness checks, the results of which are reported in the supplement. We tested whether the effects of interest were robust regardless of the year of data collection, the difference in attitude measures between Russia vs. the rest of the countries, the difference how income was operationalized in different countries (“rich” vs. “poor”, or “above average” vs. “below average”, respectively), participants’ own education and occupational status (as highly educated professionals were overrepresented in the samples), and whether effects of income were robust across occupation levels. The effects of interest were largely consistent across robustness checks, with only minor deviations when controlling for participant demographics and controlling for occupation level in income effects.

Given the small number of countries (five to eight depending on the dimension), we ran simulations with R package *simr* [137] to determine power to detect cross-level interactions. The model formula for education and gender norms, for example, was attitude ~ education* gender*GSNI + (1|participant) + (1|country). For education, we have 93% power to detect a small interaction of 0.10 with gender norms, 95% with gender inequality, and 98% with general inequality. For income, we have 93% power for gender norms, 98% for gender inequality, and 98% for general inequality. For job, we have 97% power for gender norms, 98% for gender inequality, and 98% for general inequality.

## Results

### General findings

S2 Table shows mean attitude ratings by country at each level of job, income, and education, respectively. 17.7% of variance in attitude was on the country level. Single-term deletion showed significant random slopes between countries for the interactions between gender and income (*σ²* = .089, *p* < .001), job (*σ²* = .081, *p* < .001), and education (*σ²* = .128, *p* < .001).

### Education

**Fig 3:**
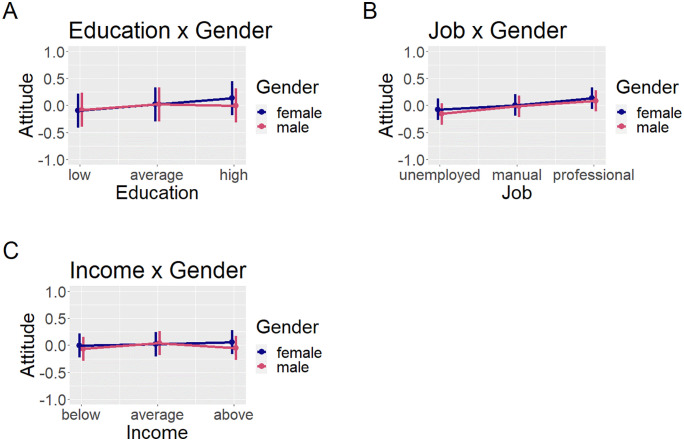
Predicted values of the two-way-interaction models. X-Axis has been cropped to better visualize interaction effects; actual range of attitude is min – 2,29, max 1.92. Error bars show 95% confidence intervals.

High education predicted more and low education less positive attitude than intermediate education. High education influenced attitudes towards men (vs. women) less positively. Table 3 shows regression results, Fig 3A visualizes the results.

**Table 3:** Regression results for education and gender predicting attitude.

Fixed component	Estimate	SE	95%CI		p
			LL	UL	
Intercept	0.02	0.16	−0.33	0.37	.909
Education high	0.12	0.02	0.08	0.15	<.001
Education low	−0.12	0.02	−0.15	−0.08	<.001
Gender male	< 0.01	0.02	−0.03	0.04	.937
Education high:gender male	−0.14	0.02	−0.19	−0.09	<.001
Education low:gender male	0.01	0.03	−0.04	0.06	.652
Random component	Variance				
Country	0.13				
Participant	0.46				
Residual	0.45				

N = 1,805, N_countries_ = 5, N_obs_ = 17,844.

**Fig 4:**
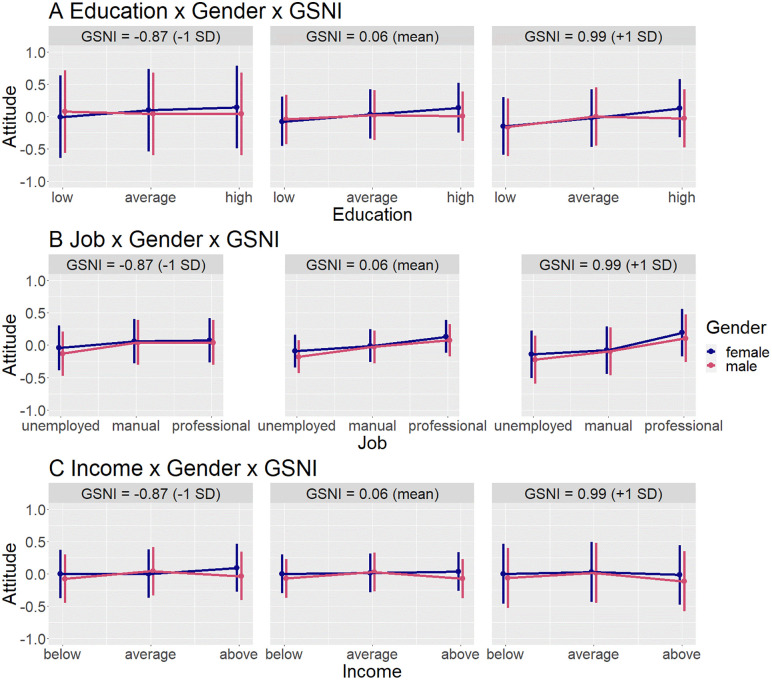
Predicted values of the three-way-interaction models including gender and gender norms (GSNI). X-Axis has been cropped to better visualize interaction effects; actual range of attitude is min – 2,29, max 1.92. Error bars show 95% confidence intervals.

In countries with more conservative gender norms, this gendered pattern was strengthened – high education influenced attitudes towards men less positively, and low education influenced attitudes towards men more negatively (about as negatively as it did for women). The education effect was mainly driven by the liking dimension of attitude, since when analyzing respect separately, it was no longer significant. See S3 Table for regression results. See Fig 4A for an interaction plot.

**Fig 5:**
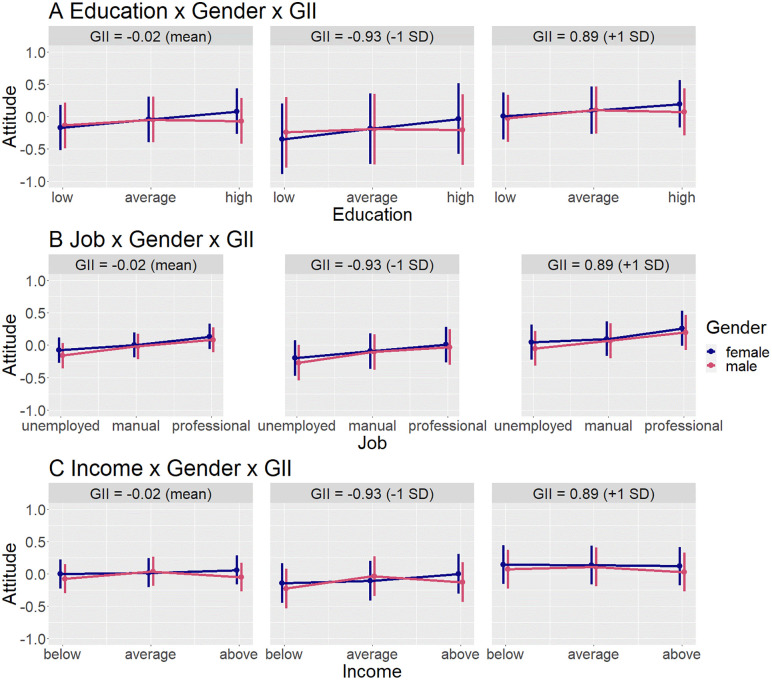
Predicted values of the three-way-interaction models including gender and gender inequality (GII). X-Axis has been cropped to better visualize interaction effects; actual range of attitude is min – 2,29, max 1.92. Error bars show 95% confidence intervals.

Similarly, in countries with high gender inequality, low education also had more negative impact on attitudes towards men. This effect was mostly driven by the respect dimension of attitude, since in a separate analysis of liking, it was only marginally significant. When analysing respect separately, there was also a positive three-way interaction with high education, indicating that high education had a more positive impact on respect towards men in countries with high gender inequality. See S6 Table for regression results. See Fig 5A for an interaction plot.

**Fig 6:**
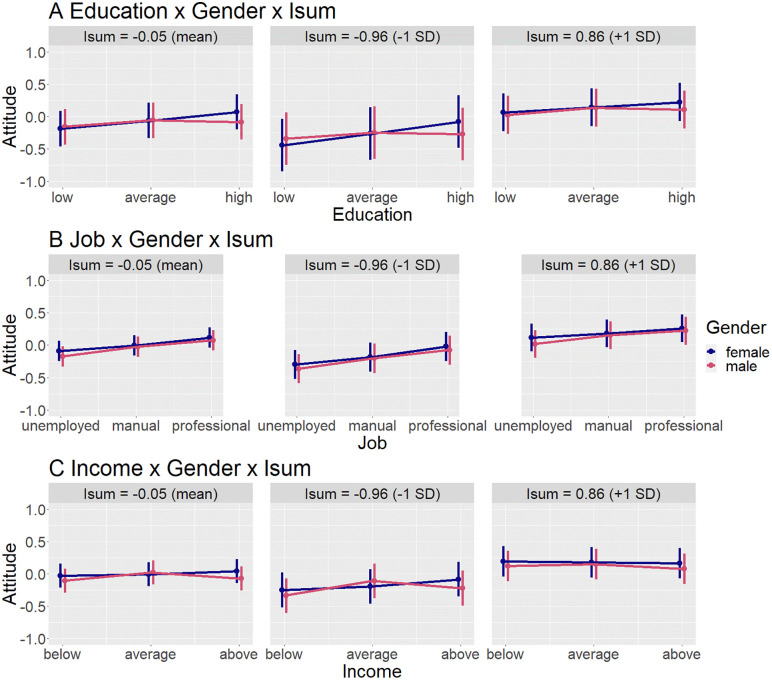
Predicted values of the three-way-interaction models including gender and general inequality (Isum). X-Axis has been cropped to better visualize interaction effects; actual range of attitude is min – 2,29, max 1.92. Error bars show 95% confidence intervals.

In highly unequal countries, low education also influenced attitudes towards men more negatively, but high education influenced attitudes towards men more positively (though still overall less positively than attitudes towards women). See S9 Table for regression results. See Fig 6A for an interaction plot.

### Occupation

Professional job predicted more and unemployment predicted less positive attitude than manual job. Unemployment had a more negative influence on attitudes towards men (vs. women). This was mostly driven by the respect dimension. When analyzing liking separately, there was no significant effect. See Table 4 for regression results and Fig 3B for a visualization.

**Table 4:** Regression results for job and gender predicting attitude*.*

Fixed component	Estimate	SE	95% CI		p
			LL	UL	
Intercept	0.03	0.10	−0.18	0.24	.793
Job professional	0.10	0.01	0.07	0.12	<.000
Job unemployed	−0.10	0.01	−0.12	−0.07	<.000
Gender male	−0.03	0.01	−0.06	−0.01	.014
Job professional:gender male	−0.01	0.02	−0.05	0.03	.654
Job unemployed:gender male	−0.05	0.02	−0.09	−0.01	.017
Random component	Variance				
Country	0.08				
Participant	0.44				
Residual	0.50				

N = 2711, N_countries_ = 8, N_obs_ = 26885.

There was no significant three-way interaction between any of the country-level variables, gender, and occupation. See S4 Table for regression results, and Fig 4B for an interaction plot with gender norms; S7 Table and Fig 5B with gender inequality; S10 Table and Fig 6B with general inequality.

### Income

High income predicted more positive attitude compared to average income. High income influenced attitudes towards men (vs. women) less positively, and low income influenced attitudes towards men (vs. women) more negatively. See Table 5 for regression results and Fig 3C for a visualization.

**Table 5:** Regression results for income and gender predicting attitude.

Fixed component	Estimate	SE	95% CI		p
			LL	UL	
Intercept	0.02	0.11	−0.22	0.26	.883
Income low	−0.02	0.02	−0.05	0.01	.158
Income high	0.04	0.02	0.01	0.07	.011
Gender male	0.02	0.02	−0.01	0.05	.251
Income low:gender male	−0.08	0.03	−0.13	−0.03	.001
Income high:gender male	−0.13	0.02	−0.18	−0.08	<.001
Random component	Variance				
Country	0.09				
Participant	0.43				
Residual	0.49				

N = 2187, N_countries_ = 7, N_obs_ = 21849.

In countries with high gender inequality, as well as high general inequality, high income had a more positive effect on attitudes towards men, and low income had a less negative effect on attitudes towards men. This effect was mostly driven by the liking dimension of attitude, since the interaction with low income was no longer significant when separately analysing respect, and the interaction with high income only marginally significant. Overall, however, high income still had less of a positive, and low income more of a negative effect, on attitudes towards men (vs. women). See S8 Table for regression results, and Fig 5C for an interaction plot with gender inequality, and S11 Table and Fig 6C with general inequality.

There was no significant three-way interaction between gender norms, gender, and income. See S5 Table for regression results, and Fig 4C for interaction plots.

## Discussion

We investigated how gender and class interact in perceptions of others in eight cultural contexts. Overall, participants rated targets with higher education, income, and occupation level more positively and those with lower education and occupation level more negatively. This aligns with people’s general preference for high social status, at least when status differences are perceived as stable and legitimate [104,138]. However, participants did not prefer average-income over low-income targets. Poor people are often stereotyped as incompetent [48], thus they may be rated less positively. However, we systematically varied education and occupational status across income groups. When participants receive explicit information about low-income targets’ education and job, they may not stereotype them as generally incompetent. Besides, attitudes towards the poor and the rich varied between countries, with more positive attitudes towards the poor and more negative attitudes towards the rich in highly unequal countries. Indeed, social inequality predicted a less attitudes towards high-status targets. Countries were selected to cover a wide range of inequality levels, so differences between highly and less unequal countries may average to a null effect of low income.

High income had a stronger positive effect on attitudes in women (vs. men), and unemployment and low income had a stronger negative effect in men (vs. women). However, high occupational status had a similar effect on attitudes towards women and men, as did low education. The finding that low status had more negative impact on attitudes towards men on two out of three dimensions mostly aligns with the Subordinate Male Target Hypothesis/SMTH [86–88,139,140], according to which men from marginalized groups, in this case low class, would face more direct negative consequences than women. Intersectional invisibility theory [87] also states that more prototypical members of marginalized groups – in this case, men – would encounter more active oppression than less prototypical members – in this case, women. Importantly, women from lower socioeconomic class backgrounds may in turn encounter distinct disadvantages related to invisibility, such as lack of support in the face of gender-based mistreatment and sexual violence [90,91] The finding also aligns with shifting standards theory, according to which minimum performance standards for women would be lower in stereotypically masculine areas like career [76], and thus low status on these dimensions would be penalized less in women. The finding could also show a backlash effect against men who violate gender norms when failing to fulfill the provider role [73]. This aligns with findings showing stricter prescriptive gender norms for men vs. women [61] and more negative stereotypes about poor men vs. women [89]. However, the lack of interaction effect with gender for education implies these theories may not hold for all status markers. Since girls/women often outperform boys/men regarding educational achievement [141] and educational effort is stereotypically associated with femininity [142,143], education standards for women may be higher compared to income and occupation. Since more material aspects of status like income are associated with masculine roles, education may be relatively more important in the evaluation of women.

The finding that contrary to our predictions, high status had a more positive effect on attitudes towards women (vs. men) on two out of three dimensions aligns with shifting standards theory – women may be assumed to be especially competent when they manage to reach high status, since participants may be aware that the hurdle for them to be considered exceptionally competent could be higher compared to men [79], as well as the necessary effort to reach the same occupational status and income as men, considering persistent gender discrimination in the workplace, and gender pay gaps. Conversely, we find no evidence for a backlash effect against women in high-status positions [65]. However, backlash effects have mostly been researched regarding organizational leadership, not necessarily more abstract social hierarchies, which may explain the lack of alignment. The labels we used in most countries suggested relatively mild status hierarchies, e.g., someone with above average income instead of a rich person; the latter may elicit stronger backlash. The finding also reflects no overall higher importance of status dimensions for attitudes towards men compared to women, contrary to what we expected based on traditional masculine stereotypes in which competence is central [60,61].

Preference patterns regarding gender or status, and their interaction, varied between countries. Conservative gender norms strengthened the general pattern that high status had less positive impact on attitudes towards men (vs. women). Conservative gender roles include a higher gender asymmetry where men are breadwinners in the public sphere, whereas women are responsible for the household [144], so status expectations for men should be higher in these contexts. In addition to normative expectation, conservative gender roles also correlate with gender inequality. Participants may therefore consider gendered differences in opportunities in these contexts and adjust their attitudes towards women and men of different status depending on the assumed difficulty to reach high status depending on gender.

High education influenced attitudes towards men less positively, and low education influenced their attitudes more negatively, when gender norms were more conservative, while gender norms did not interact with gender and job or income. This may be because men are penalized more for low income and job than women even in less conservative contexts, while that is less the case for education. Participants may also adjust their attitudes based on assumed gender differences in educational opportunities. Since conservative gender norms correlate with gender inequality, women may have less access to education in gender-conservative countries. Thus, participants may expect men to achieve higher education than women by default, and perceive them more negatively when they fail to do so.

Regarding general inequality, high education and high income influenced attitudes towards men more positively when inequality was high. This could reflect the higher importance of status in more unequal societies. Interestingly, unemployment and low income influenced attitudes towards men less negatively in highly unequal contexts, potentially reflecting participants’ awareness of low class mobility.

In countries with high gender inequality, high income had more positive and low income less negative impact on attitudes towards men. Gender inequality correlates with gender norms and general inequality, so this result may be explained by the previously suggested higher relevance of income in generally unequal contexts, especially for men who are expected to be providers when gender norms are conservative, while also accounting for low class mobility in highly inequal countries when evaluating low-income individuals.

The pattern of results was similar for overall attitude as for the specific dimensions liking and respect, but there were some nuanced differences. Gender differences in the impact of unemployment on attitude were mostly driven by respect, with unemployment impacting respect towards men more negatively than towards women. Regarding three-level interactions with gender inequality on the country level, the stronger impact of education on attitudes towards men in countries with high gender inequality was also driven by the respect dimension. The stronger positive effect of high income and weaker negative effect of low income on attitudes towards men in countries with high gender inequality on the other hand was mostly driven by the liking dimension. This could indicate that specific status markers predict some components of attitude more strongly than others, with education and occupation being more linked to respect, and income to liking. However, these findings are preliminary and need to be replicated in future studies.

Class and gender interact shaping people’s experiences, perceptions, and judgments. Gender stereotypes and role expectations differ by class [145–147], the most prominent stereotypes and norms aligned with middle-class (White, heterosexual, etc.) women and men [145,147]. Our results show bias against low-status or in favor of high-status others also varies by gender.

How these patterns play out exactly depends on social hierarchies and gender norms in a specific cultural context. Among contextual factors, these effects can be influenced by gender role expectations [148,149], importance of status [150], assumed gender differences by opportunity, as well as assumed class mobility [104], among others. This aligns with causal attribution processes, specifically augmentation [151] – for example, when contextual gender inequality would strongly suggest men to be more successful than women, there must be especially strong (likely negative, personal) explanations for a man who fails to be successful even under these circumstances. On the other hand, when contextual inequality and low social mobility suggests that some individuals would have little chance to reach high socioeconomic status no matter their efforts, people may be less likely to draw negative personal conclusions about these individuals, since their low status can be explained by the context.

### Strengths and limitations

We compared the impact of gender, class, and their interactions on interpersonal attitudes across eight countries that differed in gender role norms and inequality. This helped ensure generalizability, and identify contextual moderators. Furthermore, stimulus material in each country included people of several races/ethnicities most present in the respective local context, so that the findings apply to targets from different racial backgrounds. Our samples were demographically diverse to represent all groups included in the vignettes, including different income groups, educational backgrounds, and occupational groups. While in most countries our samples were restricted to participants we could reach via online recruitment, we made sure that groups that might be generally underrepresented in online samples, e.g., older adults, were still included. However, we did not achieve a perfectly balanced representation of all target groups. Despite all of our samples including participants from different educational and occupational backgrounds, professionals with moderate to high education levels were relatively overrepresented. While robustness checks controlling for participant education and occupation showed little impact on the results, due to the relative underrepresentation of people with lower educational/occupational status, we may not have been able to detect effects of these participant group memberships that may emerge in samples with a more balanced representation of occupational/educational groups. There might also be additional factors specific to our mostly online participants who self-selected into the study instead of being recruited at random that might limit generalizability.

The factorial survey design allowed to manipulate education, job, and income separately, while in real life, they are often intertwined [152] – for example, professional jobs generally require higher educational qualifications. Thus, we were able to identify effects specific to each factor.

However, we cannot be certain why the effects we observed occurred. Individuals may base their judgment on inferences about targets’ skills and personality that are more or less grounded in social realities and/or stereotypes – for example, if they are aware of an existing gender pay gap, they may assume that it is easier for men to reach a higher income than for women and conclude that a man with a low income must be especially incompetent. Judgments may also be influenced by perceived gender norm violation [65,66,73]. However, we did not ask about participants’ perceptions of their social environments, nor how gender-role congruent they found the targets. Beyond the specific dimension of gender, individuals with a high (situational or chronic) need for certainty tend to evaluate non-stereotypical targets more negatively because they resist easy categorization [153,154]. We did not observe an overall bias against non-stereotypical targets, as we did not observe a backlash in attitude against high-status women. Thus, our results are unlikely to stem from a general dislike of non-stereotypicality among participants. However, this mechanism might still play a role depending on participants’ personality, attitudes, and motivations, or strength and content of stereotypes about gender and class groups.

Another open question is how actual social inequality interacts with people’s beliefs about inequality in shaping attitudes towards others based on gender and status. Subjective perception of inequality better predicts well-being than objective inequality markers [116]. Meritocracy belief [155], just world belief, social dominance orientation [156], and neoliberal ideology [157] are associated with prejudice and discrimination towards low-status groups, and opposition against efforts to reduce inequality, like affirmative action [158]. The moderating effect of perceived inequality may be even stronger than that of objective inequality.

Despite the consistent patterns observed, the effect sizes in our study were small. Several factors could account for this. First, the factorial survey design allowed us to manipulate multiple dimensions simultaneously, which, while beneficial for understanding nuanced interactions, can dilute the apparent strength of any single effect. Social judgment is a complex phenomenon with many contributing factors. In this paper, we focus on information about targets’ gender, education, income, and occupational status, as well as broad indicators of cultural gender norms and inequality. However, other factors like perceivers’ own group memberships and potential ingroup bias [104], goals [159], and situational context [160] can also influence social perception and judgment. It is therefore unsurprising that the manipulated target characteristics only explain a small part of the variance in attitudes, and there is significant variation between participants. Even small effect sizes can be meaningful in social psychological research [161,162]. In large populations, small effects can have substantial cumulative impacts over time. For example, slight biases in hiring decisions can lead to significant disparities in employment rates and career advancement for different gender and class groups. Furthermore, small effects might indicate subtle, yet pervasive, forms of bias that contribute to broader social inequalities. Recognizing and addressing these small biases can cumulatively lead to important social changes and inform policies aimed at reducing inequality.

### Implications

Our findings show that dimensions of social status cannot be fully understood separately from gender, and vice versa. This highlights the importance of integrating intersectionality to accurately represent complex social structures of power and inequality [12,13]. Our findings underscore the necessity of integrating intersectionality into social policy and intervention strategies. Addressing gender and class inequalities simultaneously can provide a more comprehensive approach to reducing social injustice. Policies should be tailored to consider how class and gender intersect to influence individual experiences and societal perceptions. Additionally, promoting gender equality in conservative cultures requires nuanced strategies that address both gender norms and economic disparities. By recognizing the interconnectedness of these social dimensions, policymakers and practitioners can develop more effective interventions to combat systemic inequalities and foster inclusive social environments.

Finally, our findings add to previous research on the cost of traditional gender roles for men who fail to live up to them, which most do at some point [73,163,164]. They underscore the link between male privileges and restrictive, precarious masculinity [83]. Recognizing how gender inequalities come with a price for advantaged as well as disadvantaged groups could motivate men to get involved in fights for gender equality [165]. Highlighting the intersectional constitution of gender and class inequalities also addresses a common reaction by advantaged group members when they are confronted with their group’s privilege – they may acknowledge that in general, men for example are privileged, but state that they personally have not benefitted (much) from these privileges [166]. While in some cases, this may simply be an argument that advantaged group members use to avoid identity discomfort regardless of their actual experiences, it may also reflect real experiences of disadvantage on other identity dimensions [167]. Understanding intersectional dynamics of class, gender, and further relevant social dimensions is important to accurately reflect complex experiences of (dis)advantage depending on each individual’s combination of group memberships and their impact in different contexts. Incorporating this understanding into interventions aimed at improving intergroup relations and reducing inequalities could be a promising avenue for future research to improve the effectiveness of these interventions and reduce unwanted effects like defending, denying, or distancing oneself from privilege when confronted [166].

## Conclusion

We examined how gender and class (operationalized as education, income, and occupational status) intersect in influencing interpersonal attitudes. High education and income had a more positive impact on attitudes towards women (vs. men), and low income and occupation had a more negative impact on attitudes towards men. In gender-conservative countries, these gender differences were heightened – low educational status also had a more negative impact on attitudes towards men in these contexts. General social inequality also impacts status- and gender-based attitudes – people make weaker inferences about others based on social status and differentiate less by gender when inequality is high.

These findings highlight how gender norms interact with gender and class in shaping interpersonal attitudes. More studies on the social psychology of class and classism should take gender into account, and vice versa. This will enrich the understanding of class- and gender-based discrimination and potentially stimulate new ideas for anti-bias interventions, specifically targeting gendered class bias.

## Supporting information

S1 Table Sociodemographic characteristics of participants in each sample. (DOCX)

S2 Table Attitude ratings by country at each level of education, job, and income, respectively. (DOCX)

S3 Table Regression results for education, gender, and gender norms predicting attitude. (DOCX)

S4 Table Regression results for job, gender, and gender norms predicting attitude. (DOCX)

S5 Table Regression results for income, gender, and gender norms predicting attitude. (DOCX)

S6 Table Regression results for education, gender, and gender inequality predicting attitude. (DOCX)

S7 Table Regression results for job, gender, and gender inequality predicting attitude. (DOCX)

S8 Table Regression results for income, gender, and gender inequality predicting attitude. (DOCX)

S9 Table Regression results for education, gender, and general inequality predicting attitude. (DOCX)

S10 Table Regression results for job, gender, and general inequality predicting attitude. (DOCX)

S11 Table Regression results for income, gender, and general inequality predicting attitude. (DOCX)
