# Supplementary material for: Social judgments at the intersection of class and gender across cultures
Source: PLoS One. 2026 Feb 18;21(2):e0338029. doi: 10.1371/journal.pone.0338029 (PMC12915930; doi:10.1371/journal.pone.0338029)
Supplement: S1 Table — (DOCX) [file pone.0338029.s001.docx]

**S1 Table**

*Sociodemographic characteristics of participants in each sample*

| Dimension | | | | | | | | | | | | | | | | | | | | | | | | | | | | | | | |  | | | | | | | | | | | N | | | | % | | | | | | | |  | | | | | | | | | | | N | | % | | |  | | | | | | N | | | |  | | | | % |  |
| --- | --- | --- | --- | --- | --- | --- | --- | --- | --- | --- | --- | --- | --- | --- | --- | --- | --- | --- | --- | --- | --- | --- | --- | --- | --- | --- | --- | --- | --- | --- | --- | --- | --- | --- | --- | --- | --- | --- | --- | --- | --- | --- | --- | --- | --- | --- | --- | --- | --- | --- | --- | --- | --- | --- | --- | --- | --- | --- | --- | --- | --- | --- | --- | --- | --- | --- | --- | --- | --- | --- | --- | --- | --- | --- | --- | --- | --- | --- | --- | --- | --- | --- | --- | --- | --- | --- |
| Germany | | | | | | | | | | | | | | | | | | | | | | | | | | | | | | | |  | | | | | | | | | | |  | | | |  | | | | | | | |  | | | | | | | | | | |  | |  | | |  | | | | | |  | | | |  | | | |  |  |
| N_total_ = 301 | | | | | | | | | | | | | | | | | | | | | | | | | | | | | | | |  | | | | | | | | | | |  | | | |  | | | | | | | |  | | | | | | | | | | |  | |  | | |  | | | | | |  | | | |  | | | |  |  |
| Age in years | | | | | | | | | | | | | | | | | | | | | | | | | | | | | | | | *M* = 28.6, *SD* = 9.1 | | | | | | | | | | |  | | | |  | | | | | | | |  | | | | | | | | | | |  | |  | | |  | | | | | |  | | | |  | | | |  |  |
| Age group | | | | | | | | | | | | | | | | | | | | | | | | | | | | | | | | Young | | | | | | | | | | | 206 | | | | 68.7 | | | | | | | | Middle-aged | | | | | | | | | | | 90 | | 30.0 | | | Elderly | | | | | | 4 | | | |  | | | | 1.3 |  |
| Ethnicity | | | | | | | | | | | | | | | | | | | | | | | | | | | | | | | | German | | | | | | | | | | | 176 | | | | 57.3 | | | | | | | | Turkish | | | | | | | | | | | 58 | | 18.9 | | | Russian | | | | | | 47 | | | |  | | | | 15.3 |  |
|  | | | | | | | | | | | | | | | | | | | | | | | | | | | | | | | | Greek | | | | | | | | | | | 9.3 | | | | 3.0 | | | | | | | | Polish | | | | | | | | | | | 2 | | 0.7 | | | Syrian | | | | | | 2 | | | |  | | | | 0.7 |  |
|  | | | | | | | | | | | | | | | | | | | | | | | | | | | | | | | | Croatian | | | | | | | | | | | 1 | | | | 0.3 | | | | | | | | Other | | | | | | | | | | | 12 | | 3.9 | | |  | | | | | |  | | | |  | | | |  |  |
| Gender | | | | | | | | | | | | | | | | | | | | | | | | | | | | | | | | Male | | | | | | | | | | | 134 | | | | 42.9 | | | | | | | | Female | | | | | | | | | | | 170 | | 54.5 | | | Nonbinary | | | | | | 7 | | | |  | | | | 2.2 |  |
|  | | | | | | | | | | | | | | | | | | | | | | | | | | | | | | | | Other | | | | | | | | | | | 1 | | | | 0.3 | | | | | | | |  | | | | | | | | | | |  | |  | | |  | | | | | |  | | | |  | | | |  |  |
| Religion | | | | | | | | | | | | | | | | | | | | | | | | | | | | | | | | Not religious | | | | | | | | | | | 154 | | | | 51.3 | | | | | | | | Christian | | | | | | | | | | | 75 | | 25.0 | | | Muslim | | | | | | 54 | | | |  | | | | 18.0 |  |
|  | | | | | | | | | | | | | | | | | | | | | | | | | | | | | | | | Jewish | | | | | | | | | | | 8 | | | | 2.7 | | | | | | | | Other | | | | | | | | | | | 9 | | 3.0 | | |  | | | | | |  | | | |  | | | |  |  |
| Occupation | | | | | | | | | | | | | | | | | | | | | | | | | | | | | | | | Professional | | | | | | | | | | | 116 | | | | 38.7 | | | | | | | | Student | | | | | | | | | | | 107 | | 35.7 | | | Unemployed | | | | | | 35 | | | |  | | | | 11.7 |  |
|  | | | | | | | | | | | | | | | | | | | | | | | | | | | | | | | | Manual | | | | | | | | | | | 13 | | | | 4.3 | | | | | | | | Other | | | | | | | | | | | 29 | | 9.7 | | |  | | | | | |  | | | |  | | | |  |  |
| Income | | | | | | | | | | | | | | | | | | | | | | | | | | | | | | | | Below average | | | | | | | | | | | 133 | | | | 44.3 | | | | | | | | Average | | | | | | | | | | | 120 | | 40.0 | | | Above average | | | | | | 47 | | | |  | | | | 15.7 |  |
| Sexual orientation | | | | | | | | | | | | | | | | | | | | | | | | | | | | | | | | Heterosexual | | | | | | | | | | | 234 | | | | 78.0 | | | | | | | | Bisexual | | | | | | | | | | | 38 | | 12.7 | | | Homosexual | | | | | | 9 | | | |  | | | | 3.0 |  |
|  | | | | | | | | | | | | | | | | | | | | | | | | | | | | | | | | Asexual | | | | | | | | | | | 5 | | | | 1.7 | | | | | | | | Other | | | | | | | | | | | 14 | | 4.7 | | |  | | | | | |  | | | |  | | | |  |  |
| Migration background | | | | | | | | | | | | | | | | | | | | | | | | | | | | | | | | Non-immigrant | | | | | | | | | | | 170 | | | | 56.7 | | | | | | | | Immigrant | | | | | | | | | | | 76 | | 25.3 | | | 2nd/3rd generation immigrant | | | | | | 54 | | | |  | | | | 18.0 |  |
|  | | | | | | | | | | | | | | | | | | | | | | | | | | | | | | | |  | | | | | | | | | | |  | | | |  | | | | | | | |  | | | | | | | | | | |  | |  | | |  | | | | | |  | | | |  | | | |  |  |
| UK | | | | | | | | | | | | | | | | | | | | | | | | | | | | | | | |  | | | | | | | | | | |  | | | |  | | | | | | | |  | | | | | | | | | | |  | |  | | |  | | | | | |  | | | |  | | | |  |  |
| N_total_ = 301 | | | | | | | | | | | | | | | | | | | | | | | | | | | | | | | |  | | | | | | | | | | |  | | | |  | | | | | | | |  | | | | | | | | | | |  | |  | | |  | | | | | |  | | | |  | | | |  |  |
| Age in years | *M* = 40.2, *SD* = 16.1 | | | | | | | | | | | | |  |  | |  | | |  |  | |  | |  | |  | |  |  |  |  |  |  |  |  |  |  |  |  |  |  |  |  |  |  |  |  |  |  |  |  |  |  |  |  |  |  |  |  |  |  |  |  |  |  |  |  |  |  |  |  |  |  |  |  |  |  |  |  |  |  |  |  |  |  |
| Age group | | | | | | | | | | | | | | | | | | | | | | | | | | | | | | | | Young | | | | | | | | | | | 120 | | | | 40.3 | | | | | | | | Middle-aged | | | | | | | | | | | 144 | | 48.3 | | | Elderly | | | | | | 34 | | | |  | | | | 11.4 |  |
| Ethnicity | | | | | | | | | | | | | | | | | | | | | | | | | | | | | | | | White | | | | | | | | | | | 138 | | | | 46.3 | | | | | | | | Black | | | | | | | | | | | 79 | | 26.5 | | | Asian | | | | | | 77 | | | |  | | | | 25.8 |  |
|  | | | | | | | | | | | | | | | | | | | | | | | | | | | | | | | | Other | | | | | | | | | | | 4 | | | | 1.3 | | | | | | | |  | | | | | | | | | | |  | |  | | |  | | | | | |  | | | |  | | | |  |  |
| Gender | | | | | | | | | | | | | | | | | | | | | | | | | | | | | | | | Male | | | | | | | | | | | 136 | | | | 45.6 | | | | | | | | Female | | | | | | | | | | | 160 | | 53.7 | | | Nonbinary | | | | | | 1 | | | |  | | | | 0.3 |  |
|  | | | | | | | | | | | | | | | | | | | | | | | | | | | | | | | | Other | | | | | | | | | | | 1 | | | | 0.3 | | | | | | | |  | | | | | | | | | | |  | |  | | |  | | | | | |  | | | |  | | | |  |  |
| Religion | | | | | | | | | | | | | | | | | | | | | | | | | | | | | | | | Not religious | | | | | | | | | | | 117 | | | | 39.3 | | | | | | | | Christian | | | | | | | | | | | 110 | | 36.9 | | | Muslim | | | | | | 39 | | | |  | | | | 13.1 |  |
|  | | | | | | | | | | | | | | | | | | | | | | | | | | | | | | | | Jewish | | | | | | | | | | | 2 | | | | 0.7 | | | | | | | | Hindu | | | | | | | | | | | 12 | | 4.0 | | | Buddhist | | | | | | 6 | | | |  | | | | 2.0 |  |
|  | | | | | | | | | | | | | | | | | | | | | | | | | | | | | | | | Other | | | | | | | | | | | 12 | | | | 4.0 | | | | | | | |  | | | | | | | | | | |  | |  | | |  | | | | | |  | | | |  | | | |  |  |
| Occupation | | | | | | | | | | | | | | | | | | | | | | | | | | | | | | | | Professional | | | | | | | | | | | 147 | | | | 49.3 | | | | | | | | Unemployed | | | | | | | | | | | 47 | | 15.8 | | | Manual | | | | | | 30 | | | |  | | | | 10.1 |  |
|  | | | | | | | | | | | | | | | | | | | | | | | | | | | | | | | | Other | | | | | | | | | | | 74 | | | | 24.8 | | | | | | | |  | | | | | | | | | | |  | |  | | |  | | | | | |  | | | |  | | | |  |  |
| Income | | | | | | | | | | | | | | | | | | | | | | | | | | | | | | | | Below average | | | | | | | | | | | 124 | | | | 41.6 | | | | | | | | Average | | | | | | | | | | | 132 | | 44.3 | | | Above average | | | | | | 42 | | | |  | | | | 14.1 |  |
| Sexual orientation | | | | | | | | | | | | | | | | | | | | | | | | | | | | | | | | Heterosexual | | | | | | | | | | | 263 | | | | 88.3 | | | | | | | | Bisexual | | | | | | | | | | | 9 | | 3.0 | | | Homosexual | | | | | | 16 | | | |  | | | | 5.4 |  |
|  | | | | | | | | | | | | | | | | | | | | | | | | | | | | | | | | Asexual | | | | | | | | | | | 4 | | | | 1.3 | | | | | | | | Other | | | | | | | | | | | 6 | | 2.0 | | |  | | | | | |  | | | |  | | | |  |  |
| Migration background | | | | | | | | | | | | | | | | | | | | | | | | | | | | | | | | Non-immigrant | | | | | | | | | | | 175 | | | | 58.7 | | | | | | | | Immigrant | | | | | | | | | | | 97 | | 32.6 | | | 2nd/3rd generation immigrant | | | | | | 26 | | | |  | | | | 8.7 |  |
|  | | | | | | | | | | | | | | | | | | | | | | | | | | | | | | | |  | | | | | | | | | | |  | | | |  | | | | | | | |  | | | | | | | | | | |  | |  | | |  | | | | | |  | | | |  | | | |  |  |
| US | | | | | | | | | | | | | | | | | | | | | | | | | | | | | | | |  | | | | | | | | | | |  | | | |  | | | | | | | |  | | | | | | | | | | |  | |  | | |  | | | | | |  | | | |  | | | |  |  |
| N_total_ = 307 | | | | | | | | | | | | | | | | | | | | | | | | | | | | | | | |  | | | | | | | | | | |  | | | |  | | | | | | | |  | | | | | | | | | | |  | |  | | |  | | | | | |  | | | |  | | | |  |  |
| Age in years | *M* = 36.5, *SD* = 14.2 | | | | | | | | | | | | |  |  | |  | | |  |  | |  | |  | |  | |  |  |  |  |  |  |  |  |  |  |  |  |  |  |  |  |  |  |  |  |  |  |  |  |  |  |  |  |  |  |  |  |  |  |  |  |  |  |  |  |  |  |  |  |  |  |  |  |  |  |  |  |  |  |  |  |  |  |
| Age group | | | | | | | | | | | | | | | | | | | | | | | | | | | | | | | | Young | | | | | | | | | | | 160 | | | | 53.3 | | | | | | | | Middle-aged | | | | | | | | | | | 112 | | 37.3 | | | Elderly | | | | | | 28 | | | |  | | | | 9.3 |  |
| Ethnicity | | | | | | | | | | | | | | | | | | | | | | | | | | | | | | | | White | | | | | | | | | | | 91 | | | | 30.3 | | | | | | | | Black | | | | | | | | | | | 83 | | 27.7 | | | Asian | | | | | | 107 | | | |  | | | | 35.7 |  |
|  | | | | | | | | | | | | | | | | | | | | | | | | | | | | | | | | Hispanic | | | | | | | | | | | 9 | | | | 3.0 | | | | | | | | Other | | | | | | | | | | | 10 | | 3.3 | | |  | | | | | |  | | | |  | | | |  |  |
| Gender | | | | | | | | | | | | | | | | | | | | | | | | | | | | | | | | Male | | | | | | | | | | | 130 | | | | 43.3 | | | | | | | | Female | | | | | | | | | | | 162 | | 54.0 | | | Nonbinary | | | | | | 3 | | | |  | | | | 1.0 |  |
|  | | | | | | | | | | | | | | | | | | | | | | | | | | | | | | | | Other | | | | | | | | | | | 5 | | | | 1.7 | | | | | | | |  | | | | | | | | | | |  | |  | | |  | | | | | |  | | | |  | | | |  |  |
| Religion | | | | | | | | | | | | | | | | | | | | | | | | | | | | | | | | Not religious | | | | | | | | | | | 110 | | | | 36.7 | | | | | | | | Christian | | | | | | | | | | | 97 | | 32.3 | | | Muslim | | | | | | 64 | | | |  | | | | 21.3 |  |
|  | | | | | | | | | | | | | | | | | | | | | | | | | | | | | | | | Jewish | | | | | | | | | | | 5 | | | | 1.7 | | | | | | | | Hindu | | | | | | | | | | | 4 | | 1.3 | | | Buddhist | | | | | | 7 | | | |  | | | | 2.3 |  |
|  | | | | | | | | | | | | | | | | | | | | | | | | | | | | | | | | Other | | | | | | | | | | | 13 | | | | 4.3 | | | | | | | |  | | | | | | | | | | |  | |  | | |  | | | | | |  | | | |  | | | |  |  |
| Occupation | | | | | | | | | | | | | | | | | | | | | | | | | | | | | | | | Professional | | | | | | | | | | | 128 | | | | 42.7 | | | | | | | | Unemployed | | | | | | | | | | | 78 | | 26.0 | | | Manual | | | | | | 36 | | | |  | | | | 12.0 |  |
|  | | | | | | | | | | | | | | | | | | | | | | | | | | | | | | | | Other | | | | | | | | | | | 58 | | | | 19.3 | | | | | | | |  | | | | | | | | | | |  | |  | | |  | | | | | |  | | | |  | | | |  |  |
| Income | | | | | | | | | | | | | | | | | | | | | | | | | | | | | | | | Below average | | | | | | | | | | | 134 | | | | 44.7 | | | | | | | | Average | | | | | | | | | | | 127 | | 42.3 | | | Above average | | | | | | 39 | | | |  | | | | 13.0 |  |
| Sexual orientation | | | | | | | | | | | | | | | | | | | | | | | | | | | | | | | | Heterosexual | | | | | | | | | | | 242 | | | | 80.7 | | | | | | | | Bisexual | | | | | | | | | | | 32 | | 10.7 | | | Homosexual | | | | | | 16 | | | |  | | | | 5.3 |  |
|  | | | | | | | | | | | | | | | | | | | | | | | | | | | | | | | | Asexual | | | | | | | | | | | 4 | | | | 1.3 | | | | | | | | Other | | | | | | | | | | | 6 | | 2.0 | | |  | | | | | |  | | | |  | | | |  |  |
| Migration background | | | | | | | | | | | | | | | | | | | | | | | | | | | | | | | | Non-immigrant | | | | | | | | | | | 170 | | | | 56.7 | | | | | | | | Immigrant | | | | | | | | | | | 98 | | 32.7 | | | 2nd/3rd generation immigrant | | | | | | 32 | | | |  | | | | 10.7 |  |
|  | | | | | | | | | | | | | | | | | | | | | | | | | | | | | | | |  | | | | | | | | | | |  | | | |  | | | | | | | |  | | | | | | | | | | |  | |  | | |  | | | | | |  | | | |  | | | |  |  |
| Australia | | | | | | | | | | | | | | | | | | | | | | | | | | | | | | | |  | | | | | | | | | | |  | | | |  | | | | | | | |  | | | | | | | | | | |  | |  | | |  | | | | | |  | | | |  | | | |  |  |
| N_total_ = 359 | | | | | | | | | | | | | | | | | | | | | | | | | | | | | | | |  | | | | | | | | | | |  | | | |  | | | | | | | |  | | | | | | | | | | |  | |  | | |  | | | | | |  | | | |  | | | |  |  |
| Age in years | *M* = 36.5, *SD* = 16.0 | | | | | | | | | | | | |  |  | |  | | |  |  | |  | |  | |  | |  |  |  |  |  |  |  |  |  |  |  |  |  |  |  |  |  |  |  |  |  |  |  |  |  |  |  |  |  |  |  |  |  |  |  |  |  |  |  |  |  |  |  |  |  |  |  |  |  |  |  |  |  |  |  |  |  |  |
| Age group | | | | | | | | | | | | | | | | | | | | | | | | | | | | | | | | Young | | | | | | | | | | | 59 | | | | 16.4 | | | | | | | | Middle-aged | | | | | | | | | | | 216 | | 60.2 | | | Elderly | | | | | | 84 | | | |  | | | | 23.4 |  |
| Ethnicity | | | | | | | | | | | | | | | | | | | | | | | | | | | | | | | | European Australian | | | | | | | | | | | 267 | | | | 76.7 | | | | | | | | Asian Australian | | | | | | | | | | | 35 | | 10.1 | | | Aboriginal Australian | | | | | | 46 | | | |  | | | | 13.2 |  |
| Gender | | | | | | | | | | | | | | | | | | | | | | | | | | | | | | | | Male | | | | | | | | | | | 168 | | | | 47.1 | | | | | | | | Female | | | | | | | | | | | 189 | | 52.9 | | |  | | | | | |  | | | |  | | | |  |  |
| Religion | | | | | | | | | | | | | | | | | | | | | | | | | | | | | | | | Not religious | | | | | | | | | | | 166 | | | | 46.6 | | | | | | | | Christian | | | | | | | | | | | 171 | | 48.0 | | | Muslim | | | | | | 19 | | | |  | | | | 5.3 |  |
| Occupation | | | | | | | | | | | | | | | | | | | | | | | | | | | | | | | | Professional | | | | | | | | | | | 187 | | | | 52.1 | | | | | | | | Tradesperson | | | | | | | | | | | 163 | | 45.4 | | | Unemployed | | | | | | 9 | | | |  | | | | 2.5 |  |
| Education | University | | | | | | 119 | | | 33.1 | | | Vocational training | | | | | | | | | | | | | | | | | 171 | | 47.6 | | | | | | | High school | | | | | | 69 | | | | |  | | | 19.2 | | | | | | | | |  |  |  |  |  |  |  |  |  |  |  |  |  |  |  |  |  |  |  |  |  |  |  |  |  |
| Income | Below average | | | | | | | | | | | | | | | | | | | | | | | | | | | | | 117 | | 32.6 | | | | | | | | Average | | | | | | | | | | | | | | | | | | | | | | 188 | | | | 52.4 | | | Above average | | | | | 54 | | | |  | | | | 15.0 | | | |  |
|  |  | | | | | | | | | | | | | | | | | | | | | | | | | | | | |  | |  | | | | | | | |  | | | | | | | | | | | | | | | | | | | | | |  | | | |  | | |  | | | | |  | | | |  | | | |  | | | |  |
| Armenia |  | | | | | | | | | | | | | | | | | | | | | | | | | | | | |  | |  | | | | | | | |  | | | | | | | | | | | | | | | | | | | | | |  | | | |  | | |  | | | | |  | | | |  | | | |  | | | |  |
| N_total_ = 311 |  | | | | | | | | | | | | | | | | | | | | | | | | | | | | |  | |  | | | | | | | |  | | | | | | | | | | | | | | | | | | | | | |  | | | |  | | |  | | | | |  | | | |  | | | |  | | | |  |
| Age | Young | | | | | | | | | | | | | | | | | | | | | | | | | | | | | 122 | | 43.3 | | | | | | | | Middle-aged | | | | | | | | | | | | | | | | | | | | | | 133 | | | | 47.2 | | | Elderly | | | | | 27 | | | |  | | | | 9.6 | | | |  |
| Ethnicity | Armenian | | | | | | | | | | | | | | | | | | | | | | | | | | | | | 273 | | 87.8 | | | | | | | | Yazidi | | | | | | | | | | | | | | | | | | | | | | 17 | | | | 5.5 | | | Russian | | | | | 21 | | | |  | | | | 6.8 | | | |  |
| Gender | Male | | | | | | | | | | | | | | | | | | | | | | | | | | | | | 109 | | 35.3 | | | | | | | | Female | | | | | | | | | | | | | | | | | | | | | | 200 | | | | 64.7 | | |  | | | | |  | | | |  | | | |  | | | |  |
| Religion | Not religious | | | | | | | | | | | | | | | | | | | | | | | | | | | | | 54 | | 17.6 | | | | | | | | Christian | | | | | | | | | | | | | | | | | | | | | | 236 | | | | 76.9 | | | Yazidi | | | | | 17 | | | |  | | | | 5.5 | | | |  |
| Occupation | Professional | | | | | | | | | | | | | | | | | | | | | | | | | | | | | 215 | | 69.1 | | | | | | | | Unemployed | | | | | | | | | | | | | | | | | | | | | | 54 | | | | 17.4 | | | Manual | | | | | 42 | | | |  | | | | 13.5 | | | |  |
| Education | University | | | | | | | | 234 | | | 75.2 | | | | College | | | | | | | | | | | | | 36 | 11.6 | | High school | | | | | | | | | 41 | | | | |  | | | 13.2 | | | | | | |  |  |  |  |  |  |  |  |  |  |  |  |  |  |  |  |  |  |  |  |  |  |  |  |  |  |  |  |  |  |  |
| Income | | | | | Below average | | | | | | | | | | | | | | | | | 102 | | | | | | 32.8 | | | | | | | | | Average | | | | | | | | | | | | | | 142 | | | | | | | | 45.7 | | | | Above average | | | | | | | | | 67 | | | | | | |  | | | | 21.5 | | |  |
| Sexual orientation | | | | | Heterosexual | | | | | | | | | | | | | | | | | 289 | | | | | | 92.9 | | | | | | | | | Homosexual | | | | | | | | | | | | | | 22 | | | | | | | | 7.1 | | | |  | | | | | | | | |  | | | | | | |  | | | |  | | |  |
|  | | | | |  | | | | | | | | | | | | | | | | |  | | | | | |  | | | | | | | | |  | | | | | | | | | | | | | |  | | | | | | | |  | | | |  | | | | | | | | |  | | | | | | |  | | | |  | | |  |
| Brazil | | | | |  | | | | | | | | | | | | | | | | |  | | | | | |  | | | | | | | | |  | | | | | | | | | | | | | |  | | | | | | | |  | | | |  | | | | | | | | |  | | | | | | |  | | | |  | | |  |
| N_total_ = 282 | | | | |  | | | | | | | | | | | | | | | | |  | | | | | |  | | | | | | | | |  | | | | | | | | | | | | | |  | | | | | | | |  | | | |  | | | | | | | | |  | | | | | | |  | | | |  | | |  |
| Age | | | | | Young | | | | | | | | | | | | | | | | | 122 | | | | | | 43.3 | | | | | | | | | Middle-aged | | | | | | | | | | | | | | 133 | | | | | | | | 47.2 | | | | Elderly | | | | | | | | | 27 | | | | | | |  | | | | 9.6 | | |  |
| Ethnicity | | | | | White | | | | | | | | | | | | | | | | | 166 | | | | | | 58.9 | | | | | | | | | Black | | | | | | | | | | | | | | 25 | | | | | | | | 8.9 | | | | Mixed race | | | | | | | | | 91 | | | | | | |  | | | | 32.3 | | |  |
| Gender | | | | | Male | | | | | | | | | | | | | | | | | 140 | | | | | | 49.6 | | | | | | | | | Female | | | | | | | | | | | | | | 142 | | | | | | | | 50.4 | | | |  | | | | | | | | |  | | | | | | |  | | | |  | | |  |
| Religion | | | | | Not religious | | | | | | | | | | | | | | | | | 59 | | | | | | 20.9 | | | | | | | | | Catholic | | | | | | | | | | | | | | 122 | | | | | | | | 43.3 | | | | Evangelical | | | | | | | | | 101 | | | | | | |  | | | | 35.8 | | |  |
| Occupation | | | | | Professional | | | | | | | | | | | | | | | | | 214 | | | | | | 75.9 | | | | | | | | | Unemployed | | | | | | | | | | | | | | 7 | | | | | | | | 2.5 | | | | Manual | | | | | | | | | 61 | | | | | | |  | | | | 21.6 | | |  |
| Education | | | University | | | | | | | | 168 | | | | | | | 59.6 | | | | | | High school | | | | | | | 76 | | | | 27.0 | | | Primary school | | | | | | | | | | | | | | 38 | | | | |  | | | 13.5 | | | | |  |  |  |  |  |  |  |  |  |  |  |  |  |  |  |  |  |  |  |  |  |  |
| Income | | | | | | Below average | | | | | | | | | | | | | 52 | | | | | | | 18.4 | | | | | | | Average | | | | | | | | | | | | | | | 170 | | | | | | | | | | | | | | | | | | | 60.3 | | | Above average | | | | | 60 | | | | |  | | | | 21.3 | | |
|  | | | | | |  | | | | | | | | | | | | |  | | | | | | |  | | | | | | |  | | | | | | | | | | | | | | |  | | | | | | | | | | | | | | | | | | |  | | |  | | | | |  | | | | |  | | | |  | | |
| India | | | | | |  | | | | | | | | | | | | |  | | | | | | |  | | | | | | |  | | | | | | | | | | | | | | |  | | | | | | | | | | | | | | | | | | |  | | |  | | | | |  | | | | |  | | | |  | | |
| N_total_ = 329 | | | | | |  | | | | | | | | | | | | |  | | | | | | |  | | | | | | |  | | | | | | | | | | | | | | |  | | | | | | | | | | | | | | | | | | |  | | |  | | | | |  | | | | |  | | | |  | | |
| Age | | | | | | Young | | | | | | | | | | | | | 161 | | | | | | | 48.9 | | | | | | | Middle-aged | | | | | | | | | | | | | | | 146 | | | | | | | | | | | | | | | | | | | 44.4 | | | Elderly | | | | | 22 | | | | |  | | | | 6.7 | | |
| Ethnicity | | | | | | Bihari | | | | | | | | | | | | | 69 | | | | | | | 21.8 | | | | | | | Bengali | | | | | | | | | | | | | | | 108 | | | | | | | | | | | | | | | | | | | 34.1 | | | Tamil | | | | | 140 | | | | |  | | | | 44.2 | | |
| Gender | | | | | | Male | | | | | | | | | | | | | 201 | | | | | | | 61.3 | | | | | | | Female | | | | | | | | | | | | | | | 127 | | | | | | | | | | | | | | | | | | | 38.7 | | |  | | | | |  | | | | |  | | | |  | | |
| Religion | | | | | | Not religious | | | | | | | | | | | | | 19 | | | | | | | 5.8 | | | | | | | Hindu | | | | | | | | | | | | | | | 283 | | | | | | | | | | | | | | | | | | | 86.0 | | | Muslim | | | | | 27 | | | | |  | | | | 8.2 | | |
| Occupation | | | | | | Professional | | | | | | | | | | | | | 258 | | | | | | | 78.4 | | | | | | | Unemployed | | | | | | | | | | | | | | | 22 | | | | | | | | | | | | | | | | | | | 6.7 | | | Manual | | | | | 49 | | | | |  | | | | 14.9 | | |
| Education | | University | | | | | | 281 | | | | | | | | | | | 85.4 | | | | | | | High school | | | | | | | 33 | | | 10.0 | | | | | | Primary school | | | | | | | | | | | | | | | | 15 | | |  | | | 4.6 | | |  |  |  |  |  |  |  |  |  |  |  |  |  |  |  |  |  |  |  |  |
| Income | | | | Lower class | | | | | | | | | | | | | | | | | | | | | | | | | | | | | | 27 | | | | | | | | | | 8.2 | | | | | | | | | | Middle class | | | | | | | | | | | | | 256 | | | 77.8 | | | Upper class | | | 46 | | | |  | | | | 14.0 | | |
|  | | | |  | | | | | | | | | | | | | | | | | | | | | | | | | | | | | |  | | | | | | | | | |  | | | | | | | | | |  | | | | | | | | | | | | |  | | |  | | |  | | |  | | | |  | | | |  | | |
| Russia | | | |  | | | | | | | | | | | | | | | | | | | | | | | | | | | | | |  | | | | | | | | | |  | | | | | | | | | |  | | | | | | | | | | | | |  | | |  | | |  | | |  | | | |  | | | |  | | |
| N_total_ = 524 | | | |  | | | | | | | | | | | | | | | | | | | | | | | | | | | | | |  | | | | | | | | | |  | | | | | | | | | |  | | | | | | | | | | | | |  | | |  | | |  | | |  | | | |  | | | |  | | |
| Ethnicity | | | | Russian | | | | | | | | | | | | | | | | | | | | | | | | | | | | | | 216 | | | | | | | | | | 41.2 | | | | | | | | | | Tatar | | | | | | | | | | | | | 57 | | | 10.9 | | | Ukrainian | | | 37 | | | |  | | | | 7.1 | | |
|  | | | | Bashkir | | | | | | | | | | | | | | | | | | | | | | | | | | | | | | 95 | | | | | | | | | | 18.1 | | | | | | | | | | Armenian | | | | | | | | | | | | | 74 | | | 14.1 | | | Azerbaijani | | | 45 | | | |  | | | | 8.6 | | |
| Gender | | | | Male | | | | | | | | | | | | | | | | | | | | | | | | | | | | | | 194 | | | | | | | | | | 37.0 | | | | | | | | | | Female | | | | | | | | | | | | | 330 | | | 63.0 | | |  | | |  | | | |  | | | |  | | |
| Religion | | | | Not religious | | | | | | | | | | | | | | | | | | | | | | | | | | | | | | 100 | | | | | | | | | | 19.1 | | | | | | | | | | Christian | | | | | | | | | | | | | 240 | | | 45.8 | | | Muslim | | | 162 | | | |  | | | | 30.9 | | |
|  | | | | Other | | | | | | | | | | | | | | | | | | | | | | | | | | | | | | 22 | | | | | | | | | | 4.2 | | | | | | | | | |  | | | | | | | | | | | | |  | | |  | | |  | | |  | | | |  | | | |  | | |
| Occupation | | | | Skilled | | | | | | | | | | | | | | | | | | | | | | | | | | | | | | 469 | | | | | | | | | | 92.0 | | | | | | | | | | Low-skilled | | | | | | | | | | | | | 41 | | | 8.0 | | |  | | |  | | | |  | | | | 0.0 | | |
| Education | | | | No higher education | | | | | | | | | | | | | | | | | | | | | | | | | | | | | | 47 | | | | | | | | | | 9.0 | | | | | | | | | | Higher education | | | | | | | | | | | | | 450 | | | 85.9 | | | Doctoral degree | | | 27 | | | |  | | | | 5.2 | | |
| *Notes.* Participants were asked to indicate both their age in years, and which of the three age groups they self-identified with. | | | | | | | | | | | | | | | | | | | | | | | | | | | | | | | | | | | | | | | | | | | | | | | | | | | | | | | | | | | | | | | | | | |  |  |  |  |  |  |  |  |  |  |  |  |  |  |  |  |  |  |  |  |
