# Supplementary material for: Social judgments at the intersection of class and gender across cultures
Source: PLoS One. 2026 Feb 18;21(2):e0338029. doi: 10.1371/journal.pone.0338029 (PMC12915930; doi:10.1371/journal.pone.0338029)
Supplement: S2 Table — (DOCX) [file pone.0338029.s002.docx]

**S2 Table**

*Attitude ratings by country at each level of education, job, and income, respectively*

| Country | Education | | | | | | Job | | | | | | Income | | | | | |
| --- | --- | --- | --- | --- | --- | --- | --- | --- | --- | --- | --- | --- | --- | --- | --- | --- | --- | --- |
|  | low | | average | | high | | unemployed | | manual | | professional | | below | | average | | above | |
|  | N | M | N | M | N | M | N | M | N | M | N | M | N | M | N | M | N | M |
| Armenia | 1034 | -0.58 | 1143 | -0.62 | 933 | -0.54 | 1034 | -0.86 | 1143 | -0.52 | 933 | -0.36 | 933 | -0.71 | 1039 | -0.47 | 1138 | -0.58 |
| Australia | 1335 | -0.01 | 1077 | 0.05 | 1178 | 0.07 | 1312 | -0.09 | 1077 | 0.09 | 1201 | 0.12 | 1312 | -0.05 | 1201 | 0.08 | 1077 | 0.08 |
| Brazil | 846 | 0.39 | 746 | 0.37 | 1228 | 0.40 | 1410 | 0.37 | 846 | 0.44 | 564 | 0.37 | 940 | 0.45 | 1034 | 0.39 | 846 | 0.33 |
| Germany |  |  |  |  |  |  | 1003 | -0.16 | 998 | -0.06 | 1002 | -0.08 | 901 | -0.11 | 1054 | -0.10 | 1048 | -0.09 |
| India | 987 | 0.06 | 1304 | 0.21 | 999 | 0.25 | 1316 | 0.09 | 987 | 0.18 | 987 | 0.29 | 987 | 0.23 | 987 | 0.21 | 1316 | 0.12 |
| Russia | 1355 | -0.25 | 1756 | 0.05 | 1925 | 0.13 |  |  | 1848 | -0.22 | 3188 | 0.13 |  |  |  |  |  |  |
| UK |  |  |  |  |  |  | 1000 | -0.06 | 999 | 0.11 | 999 | 0.14 | 900 | -0.01 | 1049 | 0.07 | 1049 | 0.12 |
| US |  |  |  |  |  |  | 1012 | -0.06 | 1014 | 0.10 | 1014 | 0.08 | 913 | 0.00 | 1062 | -0.01 | 1065 | 0.11 |
