# Supplementary material for: Social judgments at the intersection of class and gender across cultures
Source: PLoS One. 2026 Feb 18;21(2):e0338029. doi: 10.1371/journal.pone.0338029 (PMC12915930; doi:10.1371/journal.pone.0338029)
Supplement: S3 Table — (DOCX) [file pone.0338029.s003.docx]

**S3 Table**

*Regression results for education, gender, and gender norms predicting attitude.*

| Step 1 |  |  |  |  |  | Step 2 |  |  |  |  |
| --- | --- | --- | --- | --- | --- | --- | --- | --- | --- | --- |
| Fixed component | Estimate | SE | 95% CI | | p | Estimate | SE | 95% CI | | p |
|  |  |  | 2.5 % | 97.5 % |  |  |  | 2.5 % | 97.5 % |  |
| (Intercept) | 0.03 | 0.20 | -0.34 | 0.41 | .883 | 0.04 | 0.20 | -0.33 | 0.42 | .839 |
| Education high | 0.11 | 0.02 | 0.07 | 0.15 | <.001 | 0.09 | 0.02 | 0.06 | 0.13 | <.001 |
| Education low | -0.10 | 0.02 | -0.13 | -0.06 | <.001 | -0.11 | 0.02 | -0.15 | -0.08 | <.001 |
| Gender male | 0.01 | 0.02 | -0.03 | 0.04 | .683 | -0.02 | 0.02 | -0.06 | 0.02 | .317 |
| GSNI | -0.04 | 0.22 | -0.44 | 0.37 | .870 | -0.07 | 0.22 | -0.47 | 0.34 | .782 |
| Education high:gender male | -0.14 | 0.02 | -0.19 | -0.09 | <.001 | -0.11 | 0.03 | -0.16 | -0.05 | <.001 |
| Education low:gender male | 0.02 | 0.03 | -0.03 | 0.06 | .552 | 0.05 | 0.03 | 0.00 | 0.11 | .050 |
| Education high:GSNI | 0.02 | 0.01 | -0.01 | 0.05 | .139 | 0.06 | 0.02 | 0.02 | 0.10 | .006 |
| Education low:GSNI | -0.06 | 0.01 | -0.09 | -0.03 | <.001 | -0.01 | 0.02 | -0.05 | 0.03 | .572 |
| Gender male:GSNI | -0.01 | 0.01 | -0.04 | 0.01 | .273 | 0.04 | 0.02 | 0.00 | 0.09 | .036 |
| Education high:gender male:GSNI |  |  |  |  |  | -0.07 | 0.03 | -0.13 | -0.01 | .015 |
| Education low:gender male:GSNI |  |  |  |  |  | -0.10 | 0.03 | -0.15 | -0.04 | .001 |
|  |  |  |  |  |  |  |  |  |  |  |
| Random component | Variance |  |  |  |  | Variance |  |  |  |  |
| Country | 0.41 |  |  |  |  | 0.41 |  |  |  |  |
| Participant | 0.68 |  |  |  |  | 0.68 |  |  |  |  |
| Residual | 0.67 |  |  |  |  | 0.67 |  |  |  |  |
| *Notes.* N = 1805, N_countries_ = 5, N_obs_ = 17844. | | | | | | | | | | |
