# Supplementary material for: Social judgments at the intersection of class and gender across cultures
Source: PLoS One. 2026 Feb 18;21(2):e0338029. doi: 10.1371/journal.pone.0338029 (PMC12915930; doi:10.1371/journal.pone.0338029)
Supplement: S4 Table — (DOCX) [file pone.0338029.s004.docx]

**S4 Table**

*Regression results for job, gender, and gender norms predicting attitude.*

|  | Step 1 |  |  |  |  | Step 2 |  |  |  |  |
| --- | --- | --- | --- | --- | --- | --- | --- | --- | --- | --- |
| Fixed component | Estimate | SE | 95% CI | | p | Estimate | SE | 95% CI | | p |
|  |  |  | LL | UL |  |  |  | LL | UL |  |
| (Intercept) | 0.02 | 0.13 | -0.22 | 0.27 | .869 | 0.02 | 0.13 | 0.17 | 5.04 | .869 |
| Job professional | 0.11 | 0.02 | 0.08 | 0.14 | <.001 | 0.11 | 0.02 | 6.91 | 21603.73 | <.001 |
| Job unemployed | -0.10 | 0.02 | -0.13 | -0.07 | <.001 | -0.10 | 0.02 | -6.31 | 21660.25 | <.001 |
| Gender male | -0.03 | 0.01 | -0.06 | 0.00 | .032 | -0.03 | 0.02 | -2.07 | 21670.39 | .038 |
| GSNI | -0.05 | 0.14 | -0.31 | 0.21 | .736 | -0.05 | 0.14 | -0.35 | 5.04 | .737 |
| Job professional:gender male | -0.03 | 0.02 | -0.07 | 0.02 | .238 | -0.03 | 0.02 | -1.18 | 21647.68 | .238 |
| Job unemployed:gender male | -0.06 | 0.02 | -0.10 | -0.01 | .013 | -0.06 | 0.02 | -2.42 | 21841.84 | .016 |
| Job professional:GSNI | 0.11 | 0.01 | 0.08 | 0.13 | <.001 | 0.11 | 0.02 | 6.74 | 21594.44 | <.001 |
| Job unemployed:GSNI | 0.01 | 0.01 | -0.01 | 0.04 | .327 | 0.00 | 0.02 | 0.28 | 21672.78 | .779 |
| Gender male:GSNI | -0.01 | 0.01 | -0.03 | 0.01 | .239 | -0.01 | 0.02 | -0.75 | 21678.86 | .454 |
| Job professional:gender male:GSNI |  |  |  |  |  | -0.01 | 0.02 | -0.59 | 21637.66 | .557 |
| Job unemployed:gender male:GSNI |  |  |  |  |  | 0.02 | 0.03 | 0.68 | 21885.92 | .498 |
|  |  |  |  |  |  |  |  |  |  |  |
| Random component | Variance |  |  |  |  | Variance |  |  |  |  |
| Country | 0.33 |  |  |  |  | 0.33 |  |  |  |  |
| Participant | 0.67 |  |  |  |  | 0.67 |  |  |  |  |
| Residual | 0.70 |  |  |  |  | 0.70 |  |  |  |  |
| *Notes.* N = 2411, N_countries_ = 7, N_obs_ = 23887. |  |  |  |  |  |  |  |  |  |  |
