# Supplementary material for: Social judgments at the intersection of class and gender across cultures
Source: PLoS One. 2026 Feb 18;21(2):e0338029. doi: 10.1371/journal.pone.0338029 (PMC12915930; doi:10.1371/journal.pone.0338029)
Supplement: S5 Table — (DOCX) [file pone.0338029.s005.docx]

**S5 Table**

*Regression results for income, gender, and gender norms predicting attitude.*

|  | Step 1 |  |  |  |  | Step 2 |  |  |  |  |
| --- | --- | --- | --- | --- | --- | --- | --- | --- | --- | --- |
| Fixed component | Estimate | SE | 95% CI | | p | Estimate | SE | 95% CI | | p |
|  |  |  | LL | UL |  |  |  | LL | UL |  |
| (Intercept) | 0.01 | 0.15 | -0.28 | 0.30 | .958 | 0.01 | 0.15 | -0.28 | 0.30 | .950 |
| Income above | -0.01 | 0.02 | -0.05 | 0.02 | .495 | -0.01 | 0.02 | -0.05 | 0.02 | .416 |
| Income below | 0.03 | 0.02 | 0.00 | 0.06 | .075 | 0.03 | 0.02 | -0.01 | 0.06 | .099 |
| Gender male | 0.02 | 0.02 | -0.02 | 0.06 | .300 | 0.02 | 0.02 | -0.02 | 0.05 | .407 |
| GSNI | 0.00 | 0.16 | -0.30 | 0.31 | .995 | 0.01 | 0.16 | -0.29 | 0.32 | .940 |
| Income above:gender male | -0.09 | 0.03 | -0.14 | -0.03 | .001 | -0.08 | 0.03 | -0.14 | -0.03 | .003 |
| Income below:gender male | -0.13 | 0.03 | -0.18 | -0.08 | <.001 | -0.13 | 0.03 | -0.18 | -0.08 | <.001 |
| Income above:GSNI | 0.00 | 0.01 | -0.02 | 0.03 | .855 | -0.01 | 0.02 | -0.05 | 0.03 | .523 |
| Income below:GSNI | -0.05 | 0.01 | -0.08 | -0.03 | <.001 | -0.07 | 0.02 | -0.11 | -0.04 | <.001 |
| Gender male:GSNI | 0.00 | 0.01 | -0.02 | 0.02 | .965 | -0.03 | 0.02 | -0.07 | 0.01 | .189 |
| Income above:gender male:GSNI |  |  |  |  |  | 0.03 | 0.03 | -0.03 | 0.09 | .277 |
| Income below:gender male:GSNI |  |  |  |  |  | 0.04 | 0.03 | -0.01 | 0.10 | .113 |
|  |  |  |  |  |  |  |  |  |  |  |
| Random component | Variance |  |  |  |  | Variance |  |  |  |  |
| Country | 0.37 |  |  |  |  | 0.36 |  |  |  |  |
| Participant | 0.66 |  |  |  |  | 0.66 |  |  |  |  |
| Residual | 0.70 |  |  |  |  | 0.70 |  |  |  |  |
| *Notes.* N = 1887, N_countries_ = 6, N_obs_ = 18851. |  |  |  |  |  |  |  |  |  |  |
