# Supplementary material for: Social judgments at the intersection of class and gender across cultures
Source: PLoS One. 2026 Feb 18;21(2):e0338029. doi: 10.1371/journal.pone.0338029 (PMC12915930; doi:10.1371/journal.pone.0338029)
Supplement: S6 Table — (DOCX) [file pone.0338029.s006.docx]

**S6 Table**

*Regression results for education, gender, and gender inequality predicting attitude.*

|  | Step 1 |  |  |  |  | Step 2 |  |  |  |  |
| --- | --- | --- | --- | --- | --- | --- | --- | --- | --- | --- |
| Fixed component | Estimate | SE | 95% CI | | p | Estimate | SE | 95% CI | | p |
|  |  |  | LL | UL |  |  |  | LL | UL |  |
| (Intercept) | -0.04 | 0.18 | -0.38 | 0.29 | .822 | -0.04 | 0.18 | -0.37 | 0.29 | .836 |
| Education high | 0.12 | 0.02 | 0.09 | 0.16 | <.001 | 0.13 | 0.02 | 0.09 | 0.16 | <.001 |
| Education low | -0.11 | 0.02 | -0.15 | -0.08 | <.001 | -0.12 | 0.02 | -0.16 | -0.09 | <.001 |
| Gender male | 0.01 | 0.02 | -0.03 | 0.04 | .745 | 0.00 | 0.02 | -0.04 | 0.03 | .930 |
| GII | 0.17 | 0.17 | -0.15 | 0.48 | .401 | 0.16 | 0.17 | -0.16 | 0.47 | .429 |
| Education high:gender male | -0.14 | 0.02 | -0.19 | -0.09 | <.001 | -0.15 | 0.03 | -0.20 | -0.10 | <.001 |
| Education low:gender male | 0.01 | 0.03 | -0.04 | 0.06 | .682 | 0.03 | 0.03 | -0.02 | 0.09 | .205 |
| Education high:GII | -0.02 | 0.01 | -0.05 | 0.00 | .104 | -0.03 | 0.02 | -0.06 | 0.01 | .114 |
| Education low:GII | 0.00 | 0.01 | -0.03 | 0.02 | .855 | 0.04 | 0.02 | 0.00 | 0.08 | .029 |
| Gender male:GII | -0.01 | 0.01 | -0.04 | 0.01 | .175 | 0.01 | 0.02 | -0.03 | 0.04 | .690 |
| Education high:gender male:GII |  |  |  |  |  | 0.02 | 0.03 | -0.03 | 0.07 | .507 |
| Education low:gender male:GII |  |  |  |  |  | -0.09 | 0.03 | -0.14 | -0.03 | .001 |
|  |  |  |  |  |  |  |  |  |  |  |
| Random component | Variance |  |  |  |  | Variance |  |  |  |  |
| Country | 0.37 |  |  |  |  | 0.37 |  |  |  |  |
| Participant | 0.68 |  |  |  |  | 0.68 |  |  |  |  |
| Residual | 0.67 |  |  |  |  | 0.67 |  |  |  |  |
| Notes. N = 1805, N_countries_ = 5, N_obs_ = 17844. | | | | | | | | | | |
