# Supplementary material for: Social judgments at the intersection of class and gender across cultures
Source: PLoS One. 2026 Feb 18;21(2):e0338029. doi: 10.1371/journal.pone.0338029 (PMC12915930; doi:10.1371/journal.pone.0338029)
Supplement: S7 Table — (DOCX) [file pone.0338029.s007.docx]

**S7 Table**

*Regression results for job, gender, and gender inequality predicting attitude.*

|  | Step 1 |  |  |  |  | Step 2 |  |  |  |  |
| --- | --- | --- | --- | --- | --- | --- | --- | --- | --- | --- |
| Fixed component | Estimate | SE | 95% CI | | p | Estimate | SE | 95% CI | | p |
|  |  |  | LL | UL |  |  |  | LL | UL |  |
| (Intercept) | 0.03 | 0.10 | -0.16 | 0.22 | .795 | 0.03 | 0.10 | -0.16 | 0.22 | .795 |
| Job professional | 0.10 | 0.01 | 0.07 | 0.13 | <.001 | 0.10 | 0.01 | 0.07 | 0.13 | <.001 |
| Job unemployed | -0.10 | 0.01 | -0.12 | -0.07 | <.001 | -0.10 | 0.01 | -0.12 | -0.07 | <.001 |
| Gender male | -0.03 | 0.01 | -0.06 | -0.01 | .014 | -0.03 | 0.01 | -0.06 | -0.01 | .015 |
| GII | 0.11 | 0.11 | -0.10 | 0.31 | .358 | 0.10 | 0.11 | -0.10 | 0.31 | .363 |
| Job professional:gender male | -0.01 | 0.02 | -0.05 | 0.03 | .576 | -0.01 | 0.02 | -0.05 | 0.03 | .572 |
| Job unemployed:gender male | -0.05 | 0.02 | -0.09 | -0.01 | .017 | -0.05 | 0.02 | -0.09 | -0.01 | .018 |
| Job professional:GII | 0.03 | 0.01 | 0.01 | 0.05 | .015 | 0.03 | 0.02 | 0.00 | 0.07 | .067 |
| Job unemployed:GII | 0.03 | 0.01 | 0.01 | 0.05 | .014 | 0.03 | 0.02 | 0.00 | 0.06 | .060 |
| Gender male:GII | -0.01 | 0.01 | -0.03 | 0.01 | .232 | -0.01 | 0.02 | -0.04 | 0.03 | .597 |
| Job professional:gender male:GII |  |  |  |  |  | 0.00 | 0.02 | -0.05 | 0.05 | .898 |
| Job unemployed:gender male:GII |  |  |  |  |  | 0.00 | 0.02 | -0.05 | 0.04 | .896 |
|  |  |  |  |  |  |  |  |  |  |  |
| Random component | Variance |  |  |  |  | Variance |  |  |  |  |
| Country | 0.28 |  |  |  |  | 0.28 |  |  |  |  |
| Participant | 0.66 |  |  |  |  | 0.66 |  |  |  |  |
| Residual | 0.71 |  |  |  |  | 0.71 |  |  |  |  |
| Notes. N = 2711, N_countries_ = 8, N_obs_ = 26885. | | | | | | | | | | |
