# Supplementary material for: Social judgments at the intersection of class and gender across cultures
Source: PLoS One. 2026 Feb 18;21(2):e0338029. doi: 10.1371/journal.pone.0338029 (PMC12915930; doi:10.1371/journal.pone.0338029)
Supplement: S8 Table — (DOCX) [file pone.0338029.s008.docx]

**S8 Table**

*Regression results for income, gender, and gender inequality predicting attitude.*

|  | Step 1 |  | |  |  |  | Step 2 |  |  |  |  |
| --- | --- | --- | --- | --- | --- | --- | --- | --- | --- | --- | --- |
| Fixed component | Estimate | SE | | 95% CI | | p | Estimate | SE | 95% CI | | p |
|  |  |  | | LL | UL |  |  |  | LL | UL |  |
| (Intercept) | 0.02 | 0.11 | | -0.20 | 0.23 | .898 | 0.01 | 0.11 | -0.20 | 0.23 | .901 |
| Income above | -0.01 | 0.02 | | -0.05 | 0.02 | .369 | -0.02 | 0.02 | -0.05 | 0.02 | .338 |
| Income below | 0.04 | 0.02 | | 0.01 | 0.07 | .010 | 0.04 | 0.02 | 0.01 | 0.07 | .008 |
| Gender male | 0.02 | 0.02 | | -0.01 | 0.05 | .222 | 0.02 | 0.02 | -0.01 | 0.06 | .214 |
| GII | 0.11 | 0.11 | | -0.11 | 0.33 | .369 | 0.13 | 0.11 | -0.08 | 0.35 | .293 |
| Income above:gender male | -0.10 | 0.03 | | -0.15 | -0.05 | <.001 | -0.10 | 0.03 | -0.15 | -0.05 | <.001 |
| Income below:gender male | -0.13 | 0.02 | | -0.17 | -0.08 | <.001 | -0.13 | 0.02 | -0.18 | -0.08 | <.001 |
| Income above:GII | 0.05 | 0.01 | | 0.03 | 0.07 | <.001 | 0.02 | 0.02 | -0.01 | 0.05 | .218 |
| Income below:GII | -0.03 | 0.01 | | -0.06 | -0.01 | .003 | -0.07 | 0.02 | -0.10 | -0.04 | <.001 |
| Gender male:GII | -0.01 | 0.01 | | -0.03 | 0.01 | .422 | -0.06 | 0.02 | -0.09 | -0.02 | .002 |
| Income above:gender male:GII |  |  | |  |  |  | 0.07 | 0.03 | 0.01 | 0.12 | .011 |
| Income below:gender male:GII |  |  | |  |  |  | 0.08 | 0.02 | 0.03 | 0.12 | .002 |
|  |  |  | |  |  |  |  |  |  |  |  |
| Random component | Variance | |  |  |  |  | Variance |  |  |  |  |
| Country | 0.30 |  | |  |  |  | 0.30 |  |  |  |  |
| Participant | 0.66 |  | |  |  |  | 0.66 |  |  |  |  |
| Residual | 0.70 |  | |  |  |  | 0.70 |  |  |  |  |
| Notes. N = 2187, Ncountries = 7, Nobs = 21849. | | | | | | | | | | | |
