# Supplementary material for: Social judgments at the intersection of class and gender across cultures
Source: PLoS One. 2026 Feb 18;21(2):e0338029. doi: 10.1371/journal.pone.0338029 (PMC12915930; doi:10.1371/journal.pone.0338029)
Supplement: S9 Table — (DOCX) [file pone.0338029.s009.docx]

**S9 Table**

*Regression results for education, gender, and general inequality predicting attitude.*

|  | Step 1 |  |  |  |  | Step 2 |  |  |  |  |
| --- | --- | --- | --- | --- | --- | --- | --- | --- | --- | --- |
| Fixed component | Estimate | SE | 95% CI | | p | Estimate | SE | 95% CI | | p |
|  |  |  | LL | UL |  |  |  | LL | UL |  |
| (Intercept) | -0.05 | 0.14 | -0.30 | 0.21 | .754 | -0.05 | 0.14 | -0.30 | 0.21 | .755 |
| Education high | 0.12 | 0.02 | 0.09 | 0.16 | <.001 | 0.13 | 0.02 | 0.10 | 0.16 | <.001 |
| Education low | -0.12 | 0.02 | -0.15 | -0.08 | <.001 | -0.13 | 0.02 | -0.16 | -0.09 | <.001 |
| Gender male | 0.00 | 0.02 | -0.03 | 0.04 | .861 | 0.00 | 0.02 | -0.03 | 0.04 | .864 |
| Inequality | 0.23 | 0.13 | -0.01 | 0.46 | .171 | 0.23 | 0.13 | -0.01 | 0.46 | .172 |
| Education high:gender male | -0.14 | 0.02 | -0.19 | -0.10 | <.001 | -0.15 | 0.03 | -0.20 | -0.11 | <.001 |
| Education low:gender male | 0.01 | 0.03 | -0.04 | 0.06 | .639 | 0.02 | 0.03 | -0.03 | 0.07 | .375 |
| Education high:inequality | -0.03 | 0.01 | -0.06 | -0.01 | .006 | -0.06 | 0.02 | -0.09 | -0.03 | .001 |
| Education low:inequality | 0.02 | 0.01 | 0.00 | 0.04 | .096 | 0.05 | 0.02 | 0.02 | 0.09 | .002 |
| Gender male:inequality | -0.01 | 0.01 | -0.03 | 0.01 | .235 | -0.01 | 0.02 | -0.04 | 0.02 | .511 |
| Education high:gender male:inequality |  |  |  |  |  | 0.05 | 0.02 | 0.01 | 0.10 | .026 |
| Education low:gender male:inequality |  |  |  |  |  | -0.06 | 0.02 | -0.11 | -0.02 | .010 |
|  |  |  |  |  |  |  |  |  |  |  |
| Random component | Variance |  |  |  |  | Variance |  |  |  |  |
| Country | 0.29 |  |  |  |  | 0.29 |  |  |  |  |
| Participant | 0.68 |  |  |  |  | 0.68 |  |  |  |  |
| Residual | 0.67 |  |  |  |  | 0.67 |  |  |  |  |
| *Notes*. N = 1805, N_countries_ = 5, N_obs_ = 17844. | | | | | | | | | | |
