# Supplementary material for: Social judgments at the intersection of class and gender across cultures
Source: PLoS One. 2026 Feb 18;21(2):e0338029. doi: 10.1371/journal.pone.0338029 (PMC12915930; doi:10.1371/journal.pone.0338029)
Supplement: S10 Table — (DOCX) [file pone.0338029.s010.docx]

**S10 Table**

*Regression results for job, gender, and general inequality predicting attitude.*

|  | Step 1 |  |  |  |  | Step 2 |  |  |  |  |
| --- | --- | --- | --- | --- | --- | --- | --- | --- | --- | --- |
| Fixed component | Estimate | SE | 95% CI | | P | Estimate | SE | 95% CI | | p |
|  |  |  | LL | UL |  |  |  | LL | UL |  |
| (Intercept) | 0.03 | 0.08 | -0.13 | 0.18 | .735 | 0.03 | 0.08 | -0.13 | 0.18 | .739 |
| Job professional | 0.09 | 0.01 | 0.06 | 0.12 | <.001 | 0.09 | 0.01 | 0.06 | 0.12 | <.001 |
| Job unemployed | -0.10 | 0.01 | -0.13 | -0.07 | <.001 | -0.10 | 0.01 | -0.13 | -0.07 | <.001 |
| Gender male | -0.03 | 0.01 | -0.06 | -0.01 | .013 | -0.03 | 0.01 | -0.06 | -0.01 | .018 |
| Inequality | 0.19 | 0.09 | 0.02 | 0.35 | .070 | 0.19 | 0.09 | 0.02 | 0.35 | .073 |
| Job professional:gender male | -0.01 | 0.02 | -0.05 | 0.04 | .788 | -0.01 | 0.02 | -0.05 | 0.04 | .792 |
| Job unemployed:gender male | -0.05 | 0.02 | -0.10 | -0.01 | .016 | -0.05 | 0.02 | -0.10 | -0.01 | .020 |
| Job professional:inequality | -0.03 | 0.01 | -0.05 | 0.00 | .044 | -0.03 | 0.02 | -0.06 | 0.01 | .117 |
| Job unemployed:inequality | 0.03 | 0.01 | 0.01 | 0.05 | .012 | 0.04 | 0.02 | 0.01 | 0.07 | .016 |
| Gender male:inequality | 0.00 | 0.01 | -0.02 | 0.02 | .683 | 0.00 | 0.02 | -0.03 | 0.04 | .867 |
| Job professional:gender male:inequality |  |  |  |  |  | 0.00 | 0.03 | -0.05 | 0.05 | .893 |
| Job unemployed:gender male:inequality |  |  |  |  |  | -0.02 | 0.02 | -0.07 | 0.03 | .397 |
|  |  |  |  |  |  |  |  |  |  |  |
| Random component | Variance |  |  |  |  | Variance |  |  |  |  |
| Country | 0.22 |  |  |  |  | 0.22 |  |  |  |  |
| Participant | 0.66 |  |  |  |  | 0.66 |  |  |  |  |
| Residual | 0.71 |  |  |  |  | 0.71 |  |  |  |  |
| Notes. N = 2711, N_countries_ = 8, N_obs_ = 26885. | | | | | | | | | | |
