# Supplementary material for: Social judgments at the intersection of class and gender across cultures
Source: PLoS One. 2026 Feb 18;21(2):e0338029. doi: 10.1371/journal.pone.0338029 (PMC12915930; doi:10.1371/journal.pone.0338029)
Supplement: S11 Table — (DOCX) [file pone.0338029.s011.docx]

**S11 Table**

*Regression results for income, gender, and general inequality predicting attitude.*

|  | Step 1 |  |  |  |  | Step 2 |  |  |  |  |
| --- | --- | --- | --- | --- | --- | --- | --- | --- | --- | --- |
| Fixed component | Estimate | SE | 95% CI | | p | Estimate | SE | 95% CI | | p |
|  |  |  | LL | UL |  |  |  | LL | UL |  |
| (Intercept) | 0.00 | 0.09 | -0.18 | 0.18 | .986 | 0.00 | 0.09 | -0.18 | 0.18 | .995 |
| Income above | -0.02 | 0.02 | -0.05 | 0.01 | .231 | -0.02 | 0.02 | -0.05 | 0.01 | .269 |
| Income below | 0.04 | 0.02 | 0.01 | 0.07 | .008 | 0.05 | 0.02 | 0.01 | 0.08 | .004 |
| Gender male | 0.02 | 0.02 | -0.01 | 0.06 | .214 | 0.03 | 0.02 | -0.01 | 0.06 | .134 |
| Inequality | 0.18 | 0.10 | 0.00 | 0.37 | .117 | 0.21 | 0.10 | 0.02 | 0.39 | .084 |
| Income above:gender male | -0.10 | 0.03 | -0.15 | -0.05 | <.001 | -0.10 | 0.03 | -0.16 | -0.05 | <.001 |
| Income below:gender male | -0.13 | 0.02 | -0.17 | -0.08 | <.001 | -0.14 | 0.02 | -0.19 | -0.09 | <.001 |
| Income above:inequality | 0.07 | 0.01 | 0.05 | 0.09 | <.001 | 0.04 | 0.02 | 0.00 | 0.07 | .037 |
| Income below:inequality | -0.03 | 0.01 | -0.05 | 0.00 | .019 | -0.07 | 0.02 | -0.10 | -0.04 | <.001 |
| Gender male:inequality | -0.01 | 0.01 | -0.03 | 0.01 | .477 | -0.06 | 0.02 | -0.10 | -0.03 | <.001 |
| Income above:gender male:inequality |  |  |  |  |  | 0.07 | 0.03 | 0.02 | 0.13 | .005 |
| Income below:gender male:inequality |  |  |  |  |  | 0.09 | 0.02 | 0.04 | 0.14 | <.001 |
|  |  |  |  |  |  |  |  |  |  |  |
| Random component | Variance |  |  |  |  | Variance |  |  |  |  |
| Country | 0.24 |  |  |  |  | 0.24 |  |  |  |  |
| Participant | 0.66 |  |  |  |  | 0.66 |  |  |  |  |
| Residual | 0.70 |  |  |  |  | 0.70 |  |  |  |  |
| Notes. N = 2187, N_countries_ = 7, N_obs_ = 21849. |  |  |  |  |  |  |  |  |  |  |
